# Supplementary material for: Understanding the language barriers to translating informed consent documents for maternal health trials in Zambia: a qualitative study
Source: BMJ Open. 2024 Apr 5;14(4):e076744. doi: 10.1136/bmjopen-2023-076744 (PMC11002372; doi:10.1136/bmjopen-2023-076744)
Supplement: Supplementary data [file bmjopen-2023-076744supp002.pdf]

Lost in translation: supplementary material

Contents

Supplementary Table 1: Content analysis of recruitment documents 2

Supplementary Table 2: Interview topic guide 5

Supplementary Table 3: Suggested terms from focus group discussions 7

Beardmore-Gray A, *et al. BMJ Open* 2024; 14:e076744. doi: 10.1136/bmjopen-2023-076744

Supplementary Table 1: Content analysis of recruitment documents

**Theme**	**Frequency (documents/13)**
**Pregnancy specific**	
Pregnancy	6
Pre-eclampsia	5
Delivery	3
Childbirth	3
Placenta	2
Complication	4
Ultrasound	3
Urine sample	3
Umbilical cord	2
Adverse birth outcomes	1
Blood pressure	2
High blood pressure	3
Pregnant women	3
High blood pressure in pregnancy	1
Contractions	1
Planned early delivery	1
Pre-term birth	1
Protein level in the urine	1
**Participation**	
Take part	7
You are being invited to take part in a research project	4
Participation/Participating	10
Withdraw	6
You are free to decline to participate	1
You are free to withdraw from the study at any time	3
You are not forced to participate in this study	2
You do not have to take part in this research if you do not wish to do so	1
Your decision will not affect the affect the care you receive in any way	4
We would like to invite you to take part	1
There is no penalty if you choose not to take part	1
You can receive care whether you take part in our study or not	1
You do not have to be in the research study to receive health care	1

We request your co-operation	1
We would like to invite you to participate in this study	1
**Voluntary Consent**	
Your participation is entirely voluntary	9
Consent	6
Consent form	7
Permission	3
Informed choice	2
I consent voluntarily as a participant in this research	1
If you agree to take part	1
**Risk**	
Concerns	5
Dangerous	2
Risk	8
Serious complications/consequences	5
Side effects	4
Discomfort	5
No risk	4
Suffer	3
Problems	6
Risk factors	1
**Benefit**	
Benefit	6
Benefit others/Benefit to society	2
Benefits to you	1
Direct benefit/may not help you directly/no personal benefit	4
Improve/Improve care/Improve health/outcomes	4
Possible/potential benefits	6
Important benefits	1
**Healthcare care related**	
Your health/Your baby's health	5
Care	4
Symptoms and signs	2
Treatments/Treating	5
Severe problems	2
Samples	3
Results	4
Medicine	3
Injection	4

Infection	3
Condition	3
Drug/study drug	2
Health	1
Healthcare providers	2
Improve care	2
Swelling	2
Test	3
Swab	2
Health facilities	1
Health information	1
Healthcare	1
**Research concepts**	
What is the purpose of the study	9
Study	11
50/50 Chance	1
Academic collaborators	1
Analysis/Analysed	1
Chance	3
Collect	7
Research	10
Published/publications	3
Knowledge	3
Measure/measurements	3
Presentation at international meetings/conference	2
Sponsor	3
Title of research	1
**Confidentiality/data management**	
Anonymous/Anonymised	4
Confidential	11
Data	5
Kept under lock and key	1
Protected	5
Information	10
Information about you	5
Results	4
Personal information/details	4
Information that is collected about you and your baby	2
Identity	4
Contact information/details	3
Identify	2
Data collected about you	1
Data collection forms	1
Data manager	1

Supplementary Table 2: Interview topic guide

Details:

**Date**	
**Start time**	
**Finish time**	
**Location**	
**Initial(s) of participant(s)**	
**Age(s)**	
**Level of education**	
**Profession**	

Notes

**Example prompts**<br><br>*What does this term/phrase mean to you?*<br><br>*What do you understand by this term/phrase?*<br><br>*How might this term be translated into Nyanja/Bemba?*<br><br>*Is there an equivalent word?*<br><br>*Is there an equivalent concept?*<br><br>**If participant is a translator:**<br><br>*Can you tell me about your experience of translating research*	

materials from English to Nyanja/Bemba?<br><br>*Have you come across any challenges?*<br><br>*Is there anything you feel that may improve this process?*<br><br>**If participant is a researcher**:<br><br>*Can you tell me about your experience of using research materials when gaining consent?*<br><br>*Have you come across any challenges?*<br><br>*Is there anything you feel that may improve this process?*	

Beardmore-Gray A, *et al. BMJ Open* 2024; 14:e076744. doi: 10.1136/bmjopen-2023-076744

Supplementary Table 3: Suggested terms from focus group discussions

English	Nyanja
Pre-eclampsia	Bvuto la kutamanga kwamagazi ndikupezeka kwa dso kudya za mthupi mumitundo pa mene muzimai ali ndi pakati
Stroke	Sitoloko
Low birthweight	Mwana opepoka
Stillborn baby	Nthayo/makanda/ana
Premature baby	Mwana osakosa/osafikapo
Swelling of the legs	Kubvimba kwa mendo
Fitting	Kukunyuka
Urine	Mkhozo/mitundo
Protein in the urine	Kupezeka kwa zakudya zamthupi mu mitundo
High blood pressure	Kutamanga Kwamagazi (Bipi)
This research is brought to you by	Atibweretsera phunziro ndi…/ Akubweretserani…
What are the risks of taking part	Ciyopyezo kuipa/ Kodi kuipa kotengaku mbali ndi kwabwanji?
Research study	Maphunzilo/ Kufunafuna/ Kufufuza
Benefit	Ubwino/Phindu
What are the benefits of taking part?	Ubwino otengako mbali ndiwabwanji?
What is the purpose of the study	Colinga caphunziro ndi ciani?
Why have I been called/invited to take part?	Ndilifukwa ciani/ndaitanidwa kuti ndi tengeko mbali?
What will happen if I take part?	Kodi ndi ciani cizacitika ndi katengaka mbali
Consent form	Cipepala cobvomekeza
Randomisation	Magulu awiri/ Komputa iza zisankila/ Magulu losadzisankhira
Healthy	Umoyo wabwino
Address	Adelesi
Analyse results	Kusanda sanda
Problem/suffering	Mabvuto
The doctor will have to induce labour	Cilikidwa kuyambisidwa

Beardmore-Gray A, *et al. BMJ Open* 2024; 14:e076744. doi: 10.1136/bmjopen-2023-076744
