# Supplementary figures and images for: Understanding the language barriers to translating informed consent documents for maternal health trials in Zambia: a qualitative study
Source: BMJ Open. 2024 Apr 5;14(4):e076744. doi: 10.1136/bmjopen-2023-076744 (PMC11002372; doi:10.1136/bmjopen-2023-076744)

Supplementary Figure 1: Theoretical framework

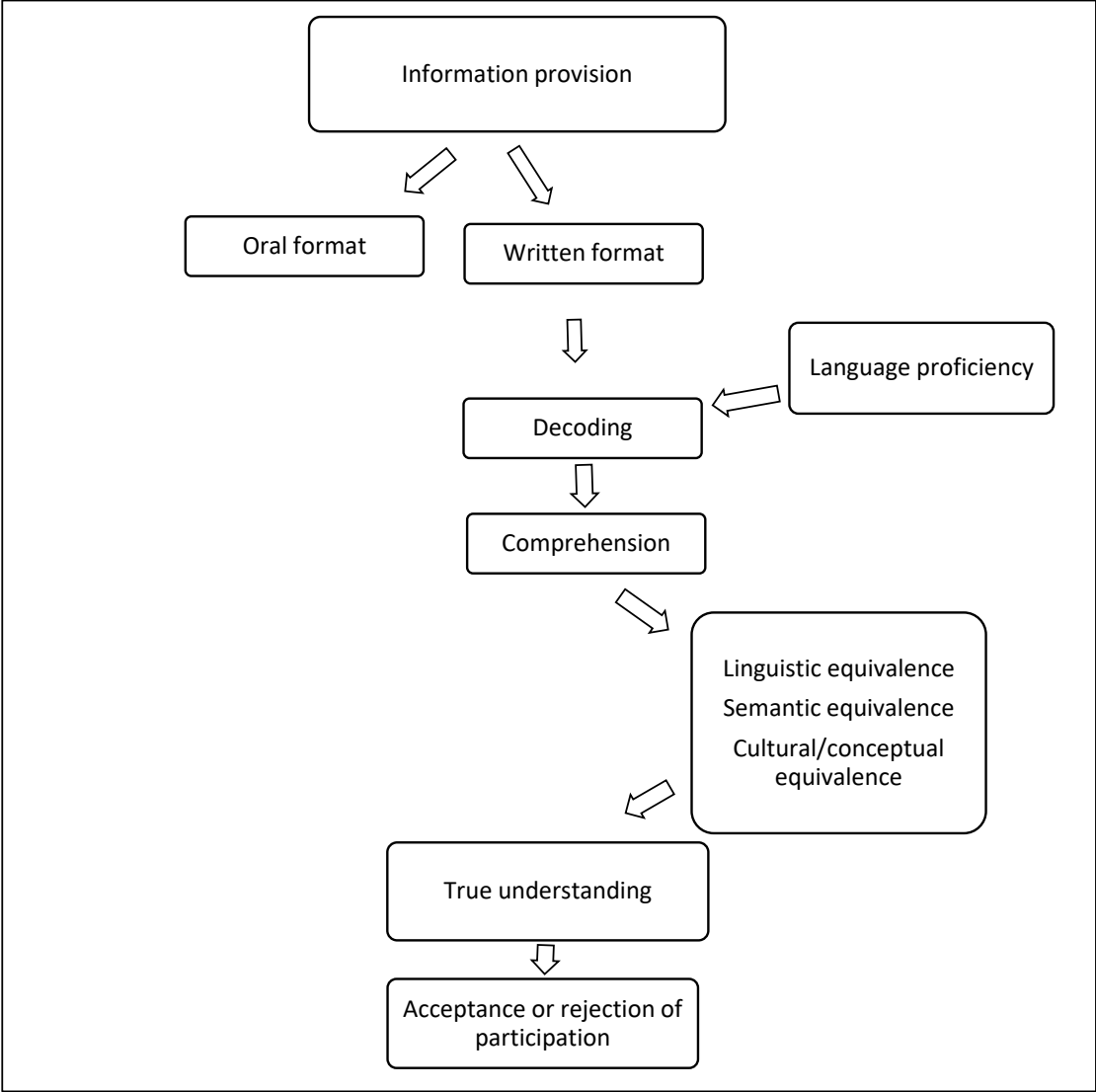

Supplement: Supplementary data [file bmjopen-2023-076744supp001.pdf]
