# Supplementary material for: Understanding the language barriers to translating informed consent documents for maternal health trials in Zambia: a qualitative study
Source: BMJ Open. 2024 Apr 5;14(4):e076744. doi: 10.1136/bmjopen-2023-076744 (PMC11002372; doi:10.1136/bmjopen-2023-076744)
Supplement: Author's manuscript [file bmjopen-2023-076744.draft_revisions.pdf]

BMJ Open

BMJ Open is committed to open peer review. As part of this commitment we make the peer review history of every article we publish publicly available.

When an article is published we post the peer reviewers' comments and the authors' responses online. We also post the versions of the paper that were used during peer review. These are the versions that the peer review comments apply to.

The versions of the paper that follow are the versions that were submitted during the peer review process. They are not the versions of record or the final published versions. They should not be cited or distributed as the published version of this manuscript.

BMJ Open is an open access journal and the full, final, typeset and author-corrected version of record of the manuscript is available on our site with no access controls, subscription charges or pay-per-view fees (http://bmjopen.bmj.com).

If you have any questions on BMJ Open's open peer review process please email info.bmjopen@bmj.com

BMJ Open

Lost in Translation: a qualitative evaluation of the barriers to informed consent in Zambia

Journal:	*BMJ Open*
Manuscript ID	bmjopen-2023-076744
Article Type:	Original research
Date Submitted by the Author:	15-Jun-2023
Complete List of Authors:	Beardmore-Gray , Alice ; King's College London, Women and Children's Health<br>Simwinga, Musonda; Zambart<br>Vwalika, Bellington; University Teaching Hospital<br>Chinkoyo, Sebastian; Ndola Teaching Hospital, Department of Obstetrics and Gynaecology<br>Chappell, Dr Lucy; King's College London, Department of Women and Children's Health, School of Life Course Sciences<br>Sandall, Jane; King's College London, Department of Women and Children's Health<br>Shennan, Andrew; King's College London, Department of Women and Children's Health, School of Life Course Sciences
Keywords:	OBSTETRICS, MEDICAL ETHICS, QUALITATIVE RESEARCH

SCHOLARONE™
Manuscripts

I, the Submitting Author has the right to grant and does grant on behalf of all authors of the Work (as defined in the below author licence), an exclusive licence and/or a non-exclusive licence for contributions from authors who are: i) UK Crown employees; ii) where BMJ has agreed a CC-BY licence shall apply, and/or iii) in accordance with the terms applicable for US Federal Government officers or employees acting as part of their official duties; on a worldwide, perpetual, irrevocable, royalty-free basis to BMJ Publishing Group Ltd ("BMJ") its licensees and where the relevant Journal is co-owned by BMJ to the co-owners of the Journal, to publish the Work in this journal and any other BMJ products and to exploit all rights, as set out in our licence.

The Submitting Author accepts and understands that any supply made under these terms is made by BMJ to the Submitting Author unless you are acting as an employee on behalf of your employer or a postgraduate student of an affiliated institution which is paying any applicable article publishing charge ("APC") for Open Access articles. Where the Submitting Author wishes to make the Work available on an Open Access basis (and intends to pay the relevant APC), the terms of reuse of such Open Access shall be governed by a Creative Commons licence – details of these licences and which Creative Commons licence will apply to this Work are set out in our licence referred to above.

Other than as permitted in any relevant BMJ Author's Self Archiving Policies, I confirm this Work has not been accepted for publication elsewhere, is not being considered for publication elsewhere and does not duplicate material already published. I confirm all authors consent to publication of this Work and authorise the granting of this licence.

For peer review only

LOST IN TRANSLATION: A QUALITATIVE EVALUATION OF THE BARRIERS TO INFORMED CONSENT IN ZAMBIA

Alice Beardmore-Gray[a], Musonda Simwinga[b], Bellington Vwalika[c], Sebastian Chinkoyo[d], Lucy C Chappell[a], Jane Sandall[a], Andrew H Shennan[a]

Author affiliations

[a]Department of Women and Children's Health, School of Life Course Sciences, King's College London, London, UK (alice.1.beardmore-gray@kcl.ac.uk, lucy.chappell@kcl.ac.uk, jane.sandall@kcl.ac.uk, andrew.shennan@kcl.ac.uk)

[b]Zambart, Lusaka, Zambia (musonda@zambart.org.zm)

[c]Department of Obstetrics and Gynaecology, University of Zambia, Lusaka, Zambia (vwalikab@gmail.com)

[d]Department of Obstetrics and Gynaecology, Ndola Teaching Hospital, Ndola, Zambia (seb.chinkoyo@yahoo.com)

Correspondence: alice.1.beardmore-gray@kcl.ac.uk

JS and AS are joint senior authors.

For peer review only

For peer review only - http://bmjopen.bmj.com/site/about/guidelines.xhtml

Abstract

Introduction

The provision of comprehensible information is essential to the process of valid informed consent. Recruitment materials designed by sponsoring institutions in English-speaking, high-income countries are commonly translated for use in global health studies in other countries; however, key concepts are often missed, misunderstood or “lost in translation”.

Methods

We explored the barriers to informed consent, focusing on the challenges of translating recruitment materials for maternal health studies into Zambian languages, using a qualitative approach. This incorporated a multi-stakeholder workshop, in-depth interviews with researchers and translators, and two community-based focus groups with volunteers from community advisory boards.

Results

The workshop highlighted difficulties in translating research terms and pregnancy-specific terms, as well as widespread concern that current templates are too long, use overly formal language, and are designed with little input from local teams. Framework analysis of in-depth interviews identified barriers to participant understanding relating to design and development of recruitment materials, local context, and communication styles. Focus group participants confirmed these findings and suggested potential solutions to ensure the language and content of recruitment materials can be better understood.

Conclusion

Our findings demonstrate that the way in which recruitment materials are currently designed, translated, and disseminated, does not enable potential participants to fully understand the

For peer review only

38 information provided. Instead of using overly complex institutional templates, recruitment materials should be created through an iterative and interactive process that provides truly comprehensible information in a format appropriate for its intended participants.

For peer review only

For peer review only - http://bmjopen.bmj.com/site/about/guidelines.xhtml

Strengths and limitations

- This study is strengthened by the use of different qualitative methods (interviews and focus groups) to collect data from different sources, thereby enhancing the reliability of our findings and ensuring a broad range of perspectives are represented
- Inclusion of additional stakeholders, such as members of ethical review boards, could have provided further information on the issue of informed consent, particularly regarding the process of ethical review of recruitment materials
- The inclusion of members of two different community advisory boards within Lusaka, Zambia, strengthens our study by providing an important community voice; however, a wider range of individuals from the community, including those more likely to be marginalised, could have ensured wider representation and added further to our findings

For peer review only

Introduction

Statement of the problem

Global health research typically involves partnerships between high income and low or middle income countries. These partnerships can sometimes perpetrate inherent structural inequalities or power dynamics,[1-5] whereby research methodology and institutional processes designed in a high income country may be imposed on low income partners without considering the relevance or acceptability to the local population. The process of informed consent, and ethical review of consent documents, are two of the domains which may be affected by this imbalance. This study evaluates an example from Lusaka, Zambia, which specifically explores how language barriers, and issues surrounding translation of recruitment materials, may impact upon informed consent. Informed consent is fundamental to any research involving human beings. For consent to be valid, participants must have the capacity to consent, act voluntarily, and be provided with sufficient comprehensible information. These principles are well described and upheld by international ethical and legal frameworks.[6,7] However, these frameworks are based upon knowledge systems generated and perpetuated by dominant groups in high-income countries[1] and often imposed upon other communities without considering local expertise. The participant information leaflets and consent forms (recruitment materials) used for enrolling participants in global health studies are often designed by sponsoring institutions based in high-income countries[8] and therefore meet the needs of trial sponsors and ethical review boards, rather than those of the intended participants. There is a focus on written documentation, complex medico-legal language and lengthy forms providing excessive information. These forms are then translated via a process of forward and back translation, into the local language(s) of the country where the research is taking place. However, a 2014 review into participant comprehension found that the majority of trial participants across different African countries did not understand several key domains of informed consent such as voluntariness, confidentiality, and the difference between taking part in research and seeking

For peer review only

medical care.[9] This is attributed to a lack of conceptual equivalence,[10-13] arising from a lack of directly equivalent terms, as well as languages that are predominantly spoken and therefore do not have standardised written formats. Use of overly complex words and medical terminology further exacerbates this lack of understanding.[14] Studies have also highlighted a lack of universal tools for assessing understanding of trial participants[9,15-17] and this in itself presents a barrier to identifying areas for improvement. Several studies have highlighted linguistic factors as a significant barrier to comprehension, but there is very little literature exploring this particular issue. Maternal health is a key research priority which justifiably attracts large numbers of research studies. However, pregnant women are a vulnerable population and in many low- or middle-income countries, including Zambia, this may be compounded by low levels of educational attainment and literacy.[18] By exploring the language barriers to cross-cultural adaptation of recruitment materials we aim to improve the quality of recruitment materials provided to future participants in maternal health studies in Zambia, and to contribute towards local efforts to strengthen research ethics capacity, which has been identified as a key priority by the Zambia National Health Research Policy and the Zambian National Health Regulatory Authority.[19]

Research objective

The overall aim of this study is to understand the language barriers to informed consent, and to demonstrate, via the example of translating maternal health research materials in Zambia, the importance of developing informed consent processes and providing participant information in a way that truly suits the needs of research participants.

Methods

We used a qualitative study design incorporating a participatory workshop, in-depth interviews, and focus group discussions. This study took place in three phases (Table 1), based primarily in Lusaka,

For peer review only - http://bmjopen.bmj.com/site/about/guidelines.xhtml

100 Zambia, alongside a timing of delivery in pre-eclampsia trial;[20] the research was led by the coordinator of this trial, a UK doctor. An SRQR checklist is provided in the supplementary materials.

For peer review only

For peer review only - http://bmjopen.bmj.com/site/about/guidelines.xhtml

Table 1: study phases and participants

Phase	Activity	Participant summary
Phase 1 Lusaka 18th Nov 2019	Workshop with invited participants from a variety of professional backgrounds. We set out to explore how key maternal health research terms, identified from a cross-sectional sample of recruitment materials, from different research studies, might be translated from English into Nyanja and Bemba and how this process might alter their meaning, as part of an initial exploratory exercise to guide the subsequent two phases	There were 11 participants including ABG (study lead). Five participants were female, and six were male. Nine were Zambian, two were British. Four were Obstetric researchers, three were research assistants and four were translators with a background in teaching and social science. Participants were invited based on their ongoing involvement with a clinical trial evaluating timing of delivery in pre-eclampsia
Phase 2 13th May – 1st July 2021	In depth interviews with key informants to understand in more detail the challenges involved with translating consent documents for a Zambian population	A total of eight interviews took place. The age range of participants was 30 to 69, three were female and five were male. Most (six) had degrees, two had diplomas. Their occupations included language teacher (three participants), research coordinator (four participants) and one community engagement officer
Phase 3 21st and 29th June 2021	Focus group discussions with local community advisory boards at primary health clinics to interrogate findings from Phases 1 and 2 with individuals who would be representative of potential research participants	A total of two focus group discussions (20 participants in total, ten in each group) took place. The mean age of participants was 28 years, twelve were female and six were male (information not provided for two participants). Eight participants had attended tertiary level education, with the remainder having attended secondary level education

Sampling strategy and data collection methods

The cross-sectional sample of recruitment materials used during Phase One was obtained by inviting researchers working in Zambia, and neighbouring countries, to submit English language examples of recruitment materials they had previously developed (and translated) to inform individuals considering participation in their research studies (predominantly clinical trials). Researchers were identified via ongoing research being conducted at University Teaching Hospital, Lusaka, ongoing research conducted by the Department of Women and Children's Health at King's College London, and via the Global Women's Research Society international conference. Researchers were asked to provide examples of participant information leaflets which were collated, read, and analysed by the

For peer review only - http://bmjopen.bmj.com/site/about/guidelines.xhtml

study lead (ABG). All relevant examples of recruitment materials provided were included in the sample. Summative content analysis (see Data analysis section) was used to identify the most commonly occurring terms related to research and pregnancy (details shown in Supplementary Table 1). Phase Two in-depth interviews were significantly delayed due to the Covid-19 pandemic. An initial convenience sample of key informants was used, whereby individuals were invited to participate if they had prior experience of either translating recruitment materials or enrolling participants into research studies. As data collection continued, additional informants were invited to participate via snowball sampling. This comprised inviting other individuals, suggested by key informants, who were likely to have relevant insight and expertise, such as community engagement officers or research assistants. Interviews were conducted in English, the working language in Zambia, by the study lead (ABG). A semi-structured interview guide was used (Supplementary Table 2) and each interview was audio recorded and then transcribed. The interviews took place at times and locations convenient to the participants, primarily office spaces and meeting venues in Lusaka, Zambia. Phase Three focus group discussions were facilitated by three of the professional language teachers/translators who had participated in the interview phase, supported by the study lead (ABG). Focus group participants were invited by asking community advisory board (CAB) members to participate if they wished. CAB members were volunteers from the local community who were part of pre-existing community groups, linked to primary healthcare facilities in Lusaka, Zambia. CAB members linked to Kanyama first level hospital and Chawama first level hospital were selected as these are two of the busiest primary healthcare facilities in Lusaka and both facilities had enrolled participants into the previously mentioned timing of delivery in pre-eclampsia trial. Invitations were sent out to CAB members via text message, and responding individuals were then invited to participate in a focus group discussion. Two initial focus groups were planned, as a purposeful sample, designed to interrogate findings from the workshop and key informant interviews. A focus group guide was developed following the Phase Two interviews and adapted from the interview topic guide (Supplementary Table 2). Focus group discussions took place in outdoor meeting spaces

For peer review only - http://bmjopen.bmj.com/site/about/guidelines.xhtml

attached to two first level hospitals (Kanyama and Chawama) in Lusaka, and were audio recorded and transcribed. Focus groups were conducted in a mixture of English, Nyanja and Bemba and were translated at the time of transcription by a Zambian research assistant.

Ethical considerations

Ethical approval for this study was provided by King's College London (MRSP-20/21-22350) and University of Zambia (1517-2020). Written informed consent was sought from all participants before any interviews or focus group discussions were conducted and participation in the study was entirely voluntary. Electronic copies of interview and focus group transcripts were stored on a password protected hard drive. Participants were anonymised and referred to by initials or numbers only.

Data analysis

Content analysis was chosen to analyse the sample of recruitment materials as a recognised method of rapidly identifying commonly occurring language. A summative approach was taken, identifying the number of times that pregnancy related phrases and research terms arose in the sample of recruitment materials.[21] During the Phase One workshop (conducted in English), participants discussed the interpretation of these commonly occurring terms, and explored how they might be translated into Nyanja and Bemba. No formal analysis of workshop data was performed, however it was used to inform the design of Phases Two and Three. Interview transcripts from Phase Two were uploaded to NVivo 12 for data coding. A framework analysis approach was used to analyse interview data.[22] The theory underpinning this framework was drawn from the conceptual framework for the process of obtaining informed consent outlined by Bhutta,[8] theories of reading and language proficiency,[23] and models for translation and cross-cultural adaptation such as those outlined by Brislin[24] and Flaherty et al.[25] These theories were combined into one overarching framework (Supplementary Figure 1[14,18,24,25]) which guided data analysis, and was developed further during the analysis process, informing the final thematic framework shown in Figure 1. Focus group data were

For peer review only - http://bmjopen.bmj.com/site/about/guidelines.xhtml

analysed using a simple inductive thematic analysis approach. The themes identified were compared and contrasted to findings from the interview data. By collecting data using different methods (workshop, interviews, and focus group discussions) and from different sources (e.g. research professionals and community members) we were able to triangulate our data [26] and test the validity of our findings from each phase, thereby enhancing the trustworthiness of our data. Focus group discussions with community members were chosen as a method of interrogating the findings from the workshop and interviews, and to seek differing perspectives and suggestions from individuals likely to represent potential research participants (as part of a local community linked to primary healthcare facilities involved in recruiting to clinical trials).

Role of the funding source

The funder of the study had no role in study design, data collection, data analysis, data interpretation, or writing of the report. The corresponding author had full access to all the data in the study and had final responsibility for the decision to submit for publication.

Patient and public involvement

Patients and the public were not involved in the design or implementation of this study. However, the initial phase, a multi-disciplinary workshop, incorporated individuals from a range of professional backgrounds including social scientists and language teachers in addition to clinicians and researchers. Subsequent phases involved professional translators and members of the public in the form of community advisory boards, with the overall findings and recommended actions reflecting their lived experience and perspectives.

For peer review only - http://bmjopen.bmj.com/site/about/guidelines.xhtml

Results

Phase One: initial workshop

Content analysis of 13 different recruitment documents, from different maternal health studies, identified the most frequently occurring terms across this sample, which were organised into relevant themes such as pregnancy-specific terms, research concepts and confidentiality (Supplementary Table 1). The workshop focussed on how these different terms could be translated for a Zambian population, and the potential difficulties that might be encountered when doing so. Through our discussion with workshop participants, we were able to identify which commonly occurring terms were most difficult to translate. For example, participating translators highlighted the lack of equivalent terms (in Nyanja or Bemba) for words such as “pre-eclampsia”, “proteinuria”, and “contractions”, as well as differing interpretations of words such as “research”, “benefits” and “risks”. The word “consent” itself was also raised as a term which could be interpreted differently depending on the context, with some communities being less familiar with the concept of individualised consent than others. Independently performed back translations of participant information leaflets that had already been translated by the participating translators (for the timing of delivery in pre-eclampsia trial) were also discussed, with important examples of discrepancies shown in Table 2. The translators performing these initial translations each worked as professional teachers of either Nyanja, Bemba, or both, in the public education system in Zambia, and each had at least five years’ experience of translating research documents for clinical trials. Although some of these discrepancies may be related to errors, rather than specific language barriers (for example, the addition of the word “funerals” in the first example provided), this highlights the importance of performing back translation (not always required by ethical review bodies), and allowing sufficient time for translators and researchers to meet face to face and discuss their work (an important process which, according to the translators interviewed, was frequently ignored by researchers). This interactive workshop highlighted several concerns regarding current procedures for designing and

For peer review only - http://bmjopen.bmj.com/site/about/guidelines.xhtml

209 translating research documents, and the lived experiences of the participants suggested this was a common and widespread issue. The group therefore proposed further exploration of the language barriers to adaptation of maternal health recruitment materials in Zambia via in-depth interviews, followed by community focus group discussions to develop locally driven solutions that may be generalisable to other researchers working in similar settings.

214 **Table 2: Examples of back translations**

Original (English)	*Back translation (from Nyanja translation)*
We appreciate your time and are grateful for your help. However, there will not be any financial compensation for taking part in this study. By choosing to take part in this study you will be helping us to help other women like you in the future.	We are very thankful for giving us your time and all the help that you have rendered to us. Even if things are like this, they will be no funerals of any kind because you have taken part in this research study.
If you take part in the study we will collect some personal information. This will only be used by members of the research team if they need to contact you. This information will be kept confidential. This means that only members of the research team will have access to it, and it will not be shared with anybody else. The data will be protected according to UK Data Protection Laws.	I agree that my suggestions that I will give should be made use of in this lesson (the way my suggestions have been presented) I am aware that my suggestions will be kept following the best recommended practices of keeping secrets.
CRADLE-4 Phase 1: The feasibility and acceptability of planned early delivery in pre-eclampsia in a Low and Middle-Income Setting.	Reviewing the advantages of early childhood delivery on the poor women and those at the centre of fending for their families. This is usually centered on the women with complications of swelling of feet and other body parts, excess proteins in the blood and urine and high blood pressure.
You have been invited to take part in this study because you have pre-eclampsia, but your condition does not require that your baby be delivered immediately.	Therefore, you are requested to take part in this study so as to help us get the facts regarding this matter.

215

For peer review only - http://bmjopen.bmj.com/site/about/guidelines.xhtml

Phases Two and Three: In-depth interviews and focus group discussions

The initial theoretical framework was modified throughout the coding process, with the final thematic framework used for data analysis shown in Figure 1.

Design and Development of recruitment materials

The interview participants working as research coordinators felt they were not given sufficient opportunity to contribute to the design of recruitment materials at an early stage, stating that they are often invited to review documents only after they have already been finalised and submitted to the ethics committee (Table 3, quote D1). Information leaflets were criticised as being too long and wordy, with emphasis placed on the need to present key messages more succinctly using alternative methods such as flyers, community announcements and household visits. The translation process itself was identified as a significant issue, due to an over-emphasis on literal word for word translations, rather than communicating the true meaning of the information. This issue was felt to be exacerbated by poor interactions between researchers and translators. The professional translators interviewed spoke of pressure to produce work within a tight timeframe, compounded by a lack of face-to-face meetings with researchers meaning that research principles and scientific concepts were often not thoroughly understood by the individual translating the document (Table 3, quote D2). Language itself was an important barrier, primarily due to a lack of equivalence – often there is simply no equivalent word in the local language for a particular English medical term. As a result, translators may try to explain the term using multiple words and phrases which ultimately distort or change the meaning (Table 3, quote D3). Furthermore, a clear distinction was made between "play" language and "formal" language with some translators criticising the overuse of formal language in translated documents, rendering them incomprehensible to the intended recipients who use different, more colloquial versions. Finally, the presence of multiple different languages in Zambia (72 in total) was identified as a further challenge, as most documents will be

For peer review only

translated into just a few of these languages which will be understood to varying degrees by different individuals depending on their family background and where they live.

Focus group participants also felt that information in recruitment documents should be shortened and simplified and that lengthy information relating to the sponsoring institution and data protection was not necessary. Many participants felt that verbal explanations, audio-visual aids and flip charts could enhance information provision but agreed with interview participants that written documentation was an important component of the process that should not be eliminated. Participants felt that greater emphasis needed to be placed on the voluntary nature of study participation, with statements such as "you do not have to take part if you do not want to" given greater prominence and translated clearly and directly to ensure the meaning was clear. Participants also stated that language related to funding needed to be clarified, as direct translation of the English phrasing implied possible financial incentives could be provided by taking part. Participants also provided examples of different terms that may be used to explain pregnancy or birth depending on the context, and that whilst informal terms were sometimes considered less "respectful", they were often better understood by their community (Table 3, quote D4). There was tension between some translators who wished to preserve the formal, grammatically correct version of their language as taught in schools, and focus group participants who preferred more colloquial terms. A suggested solution was using more informal terms in brackets so that both the official and colloquial terms could be presented and communicated effectively, depending on the user. Focus group participants also suggested creating a glossary of certain words at the start of any document, using local terms to explain in detail medical terms such as pre-eclampsia or proteinuria for the reader. Participants expressed specific preferences for different translations of particular words, examples of which are presented in Supplementary Table 3. Throughout, more informal versions were preferred, and alternative terms suggested which were sometimes different from the versions originally provided by translators.

For peer review only - http://bmjopen.bmj.com/site/about/guidelines.xhtml

Table 3: Illustrative quotes

Design and development	
D1.“what I noticed is that we just receive the consent, you can’t change anything in the consent”	Interview participant
D2. “There are people, some people, would have sent work, you work on their consignment, you just send back. You’ve never met face to face. They have no time to sit with you”	Interview participant
D3. “pre-eclampsia in our local language, we don’t have it, it’s not there, so a translator need to have a rich vocabulary and full understanding for you to come up with the correct translation”	Interview participant
D4. “here in Lusaka they don’t use kubeleka, but instead they say (abala) so for this word, it will be difficult for the community to understand”	Focus group participant
**Context and communication**	
C1. “you will find that some people, when they find these women who maybe can’t read on their own and they have to read for them, so you will find that most of the time, there is this issue of inadequate information being given and it will be like fast done”	Interview participant
C2. “you need to get consent from the husband and yet the pregnant woman is an adult, so they can consent on their own but they will not consent, they want consent from their husband or from their parents”	Interview participant
C3. “people need to understand, what is ultrasound, what is this machine, why are you doing this on me? What is its effect”	Interview participant
C4. “HIV, where you are doing blood draws so they would, from communities, they would, they would think you are selling their blood”	Interview participant
C5. “looking at the community where we come from, the people that read this information trust me, most of them can’t read, most of them can’t even read the local language”	Focus group participant

Context and communication

The way in which information is communicated to participants, as well as the context into which it is being delivered, was highlighted by both interview and focus group participants as an important area needing improvement. Some interview participants felt that potential participants are not given sufficient time to consider the information provided, with decisions often expected on the same day that a study is explained for the first time by research teams. Furthermore, some researchers described often needing to verbally explain recruitment materials to illiterate participants. They felt

For peer review only

that this makes it difficult to standardise the information provided to potential participants and risks potential participants receiving insufficient or even inaccurate information (Table 3, quote C1). When considering the context into which translated documents are being introduced, all of the interview participants raised the importance of the target population and the need to consider the levels of literacy, the languages used, the age and gender of potential participants (for example many pregnant women require their husband's consent before participating in any study) and also the common misconceptions that may be prevalent within that community surrounding healthcare interventions or research studies (Table 3, quotes C2 to C4). Many interview participants highlighted the fact that use of inappropriate language or poorly designed forms will compound this issue, and risks both limiting the number of potential participants enrolled into a study and undermining the validity of the informed consent of those who do decide to take part.

Focus group participants raised similar concerns, recalling having previously been given brochures or leaflets to read, and not having the time or inclination to do so. Having more in-depth discussions, with audio-visual aids, and the opportunity for further discussions to ask questions at a later date were suggested as measures that may improve participant comprehension. Consistent with interview findings, focus group participants highlighted the importance of understanding the target community and in particular mentioned the fact that, in their experience, most individuals in their community could not read the local language (Table 3, quote C5). They felt that simple information should be provided in ways that are easy to understand such as flip charts and pictures. However, the background and education of potential participants was also highlighted as an important factor to consider when choosing the most appropriate information format – with participants suggesting that in some communities, video consent may be deemed suspicious or inappropriate. Geographical region was also highlighted as important, with preferred terms changing depending on which area of the country the research is being conducted.

For peer review only

Discussion

Our collaborative workshop highlighted the discrepancies between the original English versions of recruitment materials and translated copies, as well as the difficulty in finding equivalent terms to accurately convey the intended meaning of key research concepts and medical words such as "pre-eclampsia". We identified several barriers to participant comprehension and informed consent within in-depth interview data, including a lack of time available to translators, poor literacy, and rushed interactions between researchers and potential participants. Researchers working in Zambia felt that the content and layout of recruitment materials were designed by "the owners" in English speaking countries and that they had little opportunity to influence the design or make their voices heard, with translations subsequently regarded as poor quality. In contrast to the grammatically correct, formal translations often used by professional translators, focus group participants expressed a clear preference for translated versions of recruitment materials to use more informal language, and that this should vary depending on the target population of a study. Furthermore, whilst workshop participants suggested audio-visual aids as a potential solution, interview and focus group participants felt that although they may be a helpful supplement, it was important to have hard copies of written information to refer back to and maintain trust.

Previous research on informed consent has focused primarily on identifying gaps in participants' understanding and evaluating community perceptions of research. Our findings correlate with those described by other studies, which found that there were widespread misconceptions regarding the purpose of research, the benefits and risks of taking part, and the use of research samples such as blood samples.[27,28] If the content of research documents does not address peoples' fears and beliefs (for example around blood tests or ultrasound scans) and explain in detail what is expected of participants and why, participants may base their decision on whether to participate or not on misinformation. Previous studies highlighted a need to further investigate the language barriers to effective communication about research, as well as to develop pre-tested and standardised tools

For peer review only - http://bmjopen.bmj.com/site/about/guidelines.xhtml

that can be used to explain research concepts in a way the local community can understand. However, ours is the first study to our knowledge which explores these barriers, with a focus on translating recruitment materials. We therefore build upon the issues raised by previous work, exploring the specific difficulties relating to language and conceptual equivalence in more detail, adding voices from a cross-section of individuals in Zambia, directly involved in the design and implementation of maternal health research, as well as community representatives of target populations.

There has been a call to action within the global health community to redress the systemic imbalances that are perpetuated by Eurocentric institutions and practices.[29] However, there are very few worked examples that demonstrate how these inequities may cause harm to research participants, and even fewer examples that suggest ways of dismantling these practices.[30] This study provides a practical and tangible example of ways in which researchers and ethical review boards can begin the process of change right away. A recent scoping review highlighted the financial, administrative and regulatory barriers to good quality ethical review in low and middle-income countries;[31]our study provides relevant findings that may be used to address some of these concerns. A collaborative, multidisciplinary research programme in Kenya has successfully implemented a systematic approach to translating contextualised informed consent templates, drawing on community engagement processes within their research programme, which has received positive engagement from researchers and ethics committees.[32] We present our own summary of our recommended actions for institutions, researchers, and translators in Figure 2, which represents the perspectives of the Zambian participants in this study, and could be used to inform a similar approach in a Zambian setting.

Strengths and Limitations

The initial research question and subsequent study design were influenced by the experiences of the study coordinator (ABG) when translating recruitment materials for the feasibility study informing

For peer review only - http://bmjopen.bmj.com/site/about/guidelines.xhtml

the main timing of delivery in pre-eclampsia trial,[20,33] which suggested the specific difficulties encountered during this process may represent a wider issue. This was explored further during the course of data collection and analysis, acknowledging the potential biases that may have been carried forwards from this initial experience. Collecting data from different sources helped to counteract any inherent individual bias. For example, the assumption that participants might prefer information provided in alternative formats was dispelled by bother interview and focus group participants who felt it was important to have a written, hard copy of any recruitment materials. In their position as a trial coordinator, it is possible that interview and focus group participants may have viewed them as possessing a certain level of authority, and this in turn may have influenced the responses of the participants. Steps taken to counteract this included a relaxed communication style during interviews and using local translators to help facilitate focus group discussions. In their position as a researcher based in Zambia during the time period that this study took place, the study lead was able to connect with and seek out key informants within the local research community and seek guidance from local experts working in social science research. Language teachers and translators represented an important group of participants for this research. Whilst they had previous professional experiences of translating research materials, it was clear that the objective of this study was to learn from and understand their experiences, rather than engage them in a professional capacity, thus limiting the potential for any conflict of interest. Focus group participants were invited to attend from local community advisory boards (CABs). These groups are local volunteers who are often consulted to gain community input and perspectives on healthcare interventions and research studies. Whilst this meant they were well-placed to participate in the focus group discussions facilitated as part of this study, participants outside of this well-established model may have provided a wider array of insights.

The views of both interview and focus group participants likely represent an urban population, though many interview participants had experience of a wide range of research studies conducted over different time periods and in different areas of the country. Interview and focus group

For peer review only - http://bmjopen.bmj.com/site/about/guidelines.xhtml

participants had many experiences of research, given that they lived in Lusaka, the capital city, where many of the healthcare facilities have ongoing involvement in several research projects. A more remote setting in areas where participants are less familiar with research may have provided different findings. However, given the aim of this study was to specifically explore issues when translating, using and understanding participant information documents, the selected population is likely appropriate for the research objectives. Inclusion of ethical review board members, or study principal investigators, who are responsible for approving many of the recruitment materials used in global health studies, could have added an additional and important perspective on the issues explored in our study. Engaging these key stakeholders would be important in any future research, and when implementing our recommended actions.

Conclusions

Our study has identified that current methods of designing and translating recruitment materials for potential research participants in maternal health studies in Zambia, do not always facilitate true understanding, and therefore do not serve the needs of their intended recipients. This problem requires researchers and ethics committees to re-evaluate their current practice and move away from viewing translation as merely a tick-box exercise required to gain ethical approval, but a collaborative and dynamic process that can be adapted to suit the needs of the communities, countries, and languages in which the research is taking place.

Declaration of interests

The authors declare no conflicts of interest.

Author Contributions

ABG, AS and LCC were involved in the study conception and in securing funding for the study. ABG, MS, BV, SC, LCC, JS and AS designed the study protocol and secured ethical approval for the study. ABG coordinated the study supported by BV and SC. ABG did the study analysis with input from MS

For peer review only - http://bmjopen.bmj.com/site/about/guidelines.xhtml

and JS. ABG wrote the original manuscript draft. All authors reviewed, contributed to, and approved the final version of the manuscript.

Funding

This study was funded by the King's ODA Research Partnership Seed Fund (KODA_1819_002). The funder of the study had no role in study design, data collection, data analysis, data interpretation, or writing of the report. The corresponding author had full access to all the data in the study and had final responsibility for the decision to submit for publication.

Acknowledgements

We thank all the individuals who participated this study, providing their valuable time and insight. Jane Sandall is an NIHR Senior Investigator and is supported by the National Institute for Health Research (NIHR) Applied Research Collaboration South London (NIHR ARC South London) at King's College Hospital NHS Foundation Trust. The views expressed are those of the author[s] and not necessarily those of the NIHR or the Department of Health and Social Care.

Data sharing

The dataset will be available to appropriate academic parties on request from the corresponding author in accordance with the data sharing policies of King's College London, with input from the co-author group where applicable.

For peer review only

References

1. Bhakuni H, Abimbola S. Epistemic injustice in academic global health. *Lancet Glob Health* 2021; **9**(10): e1465-e70.
2. Zarowsky C. Global health research, partnership, and equity: no more business-as-usual. *BMC Int Health Hum Rights* 2011; **11 Suppl 2**(Suppl 2): S1.
3. Rasheed MA. Navigating the violent process of decolonisation in global health research: a guideline. *Lancet Glob Health* 2021; **9**(12): e1640-e1.
4. Horton R. Offline: Challenging America's hegemony in global health. *Lancet* 2013; **382**(9890): 382-.
5. Hommes F, Monzo HB, Ferrand RA, et al. The words we choose matter: recognising the importance of language in decolonising global health. *Lancet Glob Health* 2021; **9**(7): e897-e8.
6. World Medical Association. WMA Declaration of Helskini - Ethicial Principles for Scientific Requirements and Research Protocols. 2013. https://www.wma.net/policies-post/wma-declaration-of-helsinki-ethical-principles-for-medical-research-involving-human-subjects/ (accessed 4th February 2023.
7. Council for International Organizations of Medical Sciences. International Ethical Guidelines for Health-related Research Involving Humans Fourth Edition. Geneva. 2016. p. 1-122.
8. Bhutta ZA. Beyond informed consent. *Bull World Health Organ* 2004; **82**(10): 771-7.
9. Afolabi MO, Okebe JU, McGrath N, Larson HJ, Bojang K, Chandramohan D. Informed consent comprehension in African research settings. *Trop Med Int Health* 2014; **19**(6): 625-42.
10. Oduro AR, Aborigo RA, Amugsi D, et al. Understanding and retention of the informed consent process among parents in rural northern Ghana. *BMC Med Ethics* 2008; **9**: 12.
11. Marshall PA. "Cultural competence" and informed consent in international health research. *Camb Q Healthc Ethics* 2008; **17**(2): 206-15.
12. Gjersing L, Caplehorn JR, Clausen T. Cross-cultural adaptation of research instruments: language, setting, time and statistical considerations. *BMC Med Res Methodol* 2010; **10**: 13.
13. Tekola F, Bull SJ, Farsides B, et al. Tailoring consent to context: designing an appropriate consent process for a biomedical study in a low income setting. *PLoS Negl Trop Dis* 2009; **3**(7): e482.
14. Villafranca A, Kereliuk S, Hamlin C, Johnson A, Jacobsohn E. The Appropriateness of Language Found in Research Consent Form Templates: A Computational Linguistic Analysis. *PLoS One* 2017; **12**(2): e0169143.
15. Appelbaum PS. Understanding "Understanding": An Important Step Toward Improving Informed Consent to Research. *AJOB Primary Research* 2010; **1**(2): 1-3.
16. Sand K, Kaasa S, Loge JH. The Understanding of Informed Consent Information—Definitions and Measurements in Empirical Studies. *AJOB Primary Research* 2010; **1**(2): 4-24.
17. Lindegger G, Milford C, Slack C, Quayle M, Xaba X, Vardas E. Beyond the checklist: assessing understanding for HIV vaccine trial participation in South Africa. *J Acquir Immune Defic Syndr* 2006; **43**(5): 560-6.
18. Zambia Statistics Agency, Ministry of Health Zambia, ICF International. Zambia Demographic and Health Survey 2018. Lusaka, Zambia and Rockville, Maryland, USA: Zambia Statistics Agency, Ministry of Health, and ICF.; 2019.
19. National Health Research Authority. Building capacity for research ethics and regulation in Zambia. 2020. https://www.nhra.org.zm/new-https://www.nhra.org.zm/new (accessed 31st December 2020.
20. Beardmore-Gray A, Vousden N, Charantimath U, et al. Planned early delivery versus expectant management to reduce adverse pregnancy outcomes in pre-eclampsia in a low- and middle-income setting: study protocol for a randomised controlled trial (CRADLE-4 Trial). *Trials* 2020; **21**(1): 960.
21. Hsieh H-F, Shannon SE. Three approaches to qualitative content analysis. *Qualitative health research* 2005; **15**(9): 1277-88.

For peer review only - http://bmjopen.bmj.com/site/about/guidelines.xhtml

22. Srivastava A, Thomson S. Framework analysis: a qualitative methodology for applied policy research. *Journal of Administration and Governance* 2009; **4**(2).

23. Mweli H. National framework for the teaching of reading in African languages in the foundation phase. Pretoria, Republic of South Africa: Department of Basic Education; 2020.

24. Brislin RW. Back-translation for cross-cultural research. *Journal of Cross-Cultural Psychology* 1970; **1**(3): 185-216.

25. Flaherty JA, Gaviria FM, Pathak D, et al. Developing instruments for cross-cultural psychiatric research. *J Nerv Ment Dis* 1988; **176**(5): 257-63.

26. Carter N, Bryant-Lukosius D, DiCenso A, Blythe J, Neville AJ. The use of triangulation in qualitative research. *Oncol Nurs Forum* 2014; **41**(5): 545-7.

27. Molyneux CS, Peshu N, Marsh K. Understanding of informed consent in a low-income setting: three case studies from the Kenyan Coast. *Soc Sci Med* 2004; **59**(12): 2547-59.

28. Molyneux S, Bull S. Consent and Community Engagement in Diverse Research Contexts: Reviewing and Developing Research and Practice:Participants in the Community Engagement and Consent Workshop, Kilifi, Kenya, March 2011. *Journal of Empirical Research on Human Research Ethics* 2013; **8**(4): 1-18.

29. Buyum AM, Kenney C, Koris A, Mkumba L, Raveendran Y. Decolonising global health: if not now, when? *BMJ Glob Health* 2020; **5**(8).

30. Khan M, Abimbola S, Aloudat T, Capobianco E, Hawkes S, Rahman-Shepherd A. Decolonising global health in 2021: a roadmap to move from rhetoric to reform. *BMJ Glob Health* 2021; **6**(3).

31. Chaudhry I, Thurtle V, Foday E, et al. Strengthening ethics committees for health-related research in sub-Saharan Africa: a scoping review. *BMJ Open* 2022; **12**(11): e062847.

32. Boga M, Davies A, Kamuya D, et al. Strengthening the Informed Consent Process in International Health Research through Community Engagement: The KEMRI-Wellcome Trust Research Programme Experience. *PLOS Medicine* 2011; **8**(9): e1001089.

33. Beardmore-Gray A, Vousden N, Silverio SA, et al. Planned early delivery for late preterm pre-eclampsia in a low- and middle-income setting: a feasibility study. *Reprod Health* 2021; **18**(1): 110.

For peer review only

For peer review only - http://bmjopen.bmj.com/site/about/guidelines.xhtml

Figure 1: Thematic framework

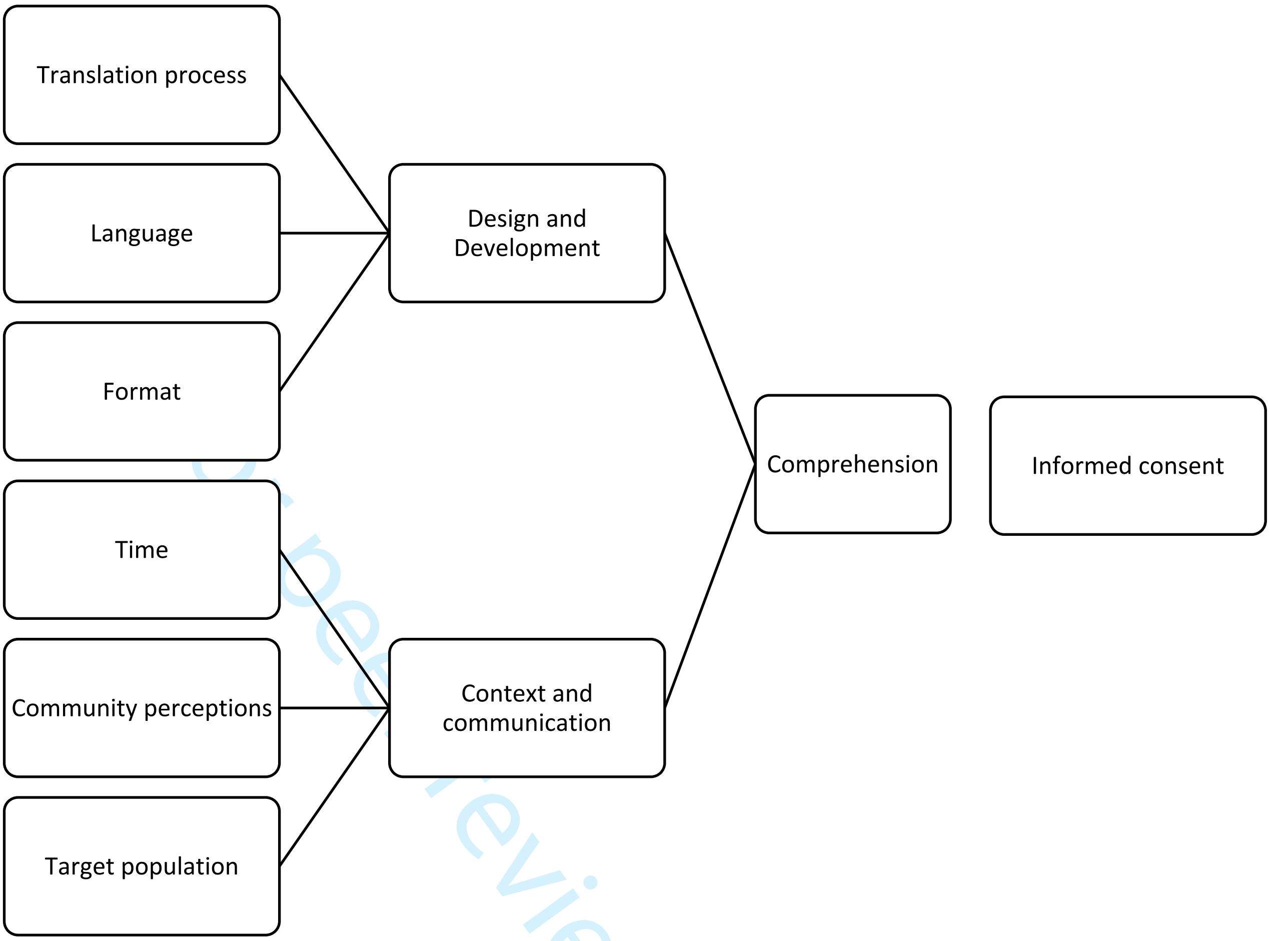

Figure 2: Summary of recommended actions

Research institutions and ethics committees

- Adopt a more flexible and adaptive approach to templates
- Suport research teams to develop recruitment materials that are context-specific
- Ensure study protocols allow sufficient time and funding to support a robust translation process and consent process including community engagement activities
- Ensure strong oversight mechanisms to verify the quality and appropriateness of translated materials
- Support further research into alternative methods of providing participant information, such as pictures and videos

Researchers

- Set aside sufficient time and funding to develop recruitment materials
- Meet face to face with translators and local language experts, ensuring the true meaning of recruitment materials can be understood
- Involve community representatives and local researchers from the outset, piloting early versions of translated materials and responding dynamically to feedback
- Move away from lengthy word documents with information that may be considered irrelevant by potential participants
- Consider a glossary of key terms at the start of any document, using simple and informal terms to explain important concepts or medical terms
- Consider the most appropriate format for the intended recipients, including flip charts and videos if appropriate

Translators

- Move away from literal, word for word translations
- Explore, and be guided by, local dialects and preferences for more informal language

For peer review only

Supplementary Figure 1: Theoretical framework

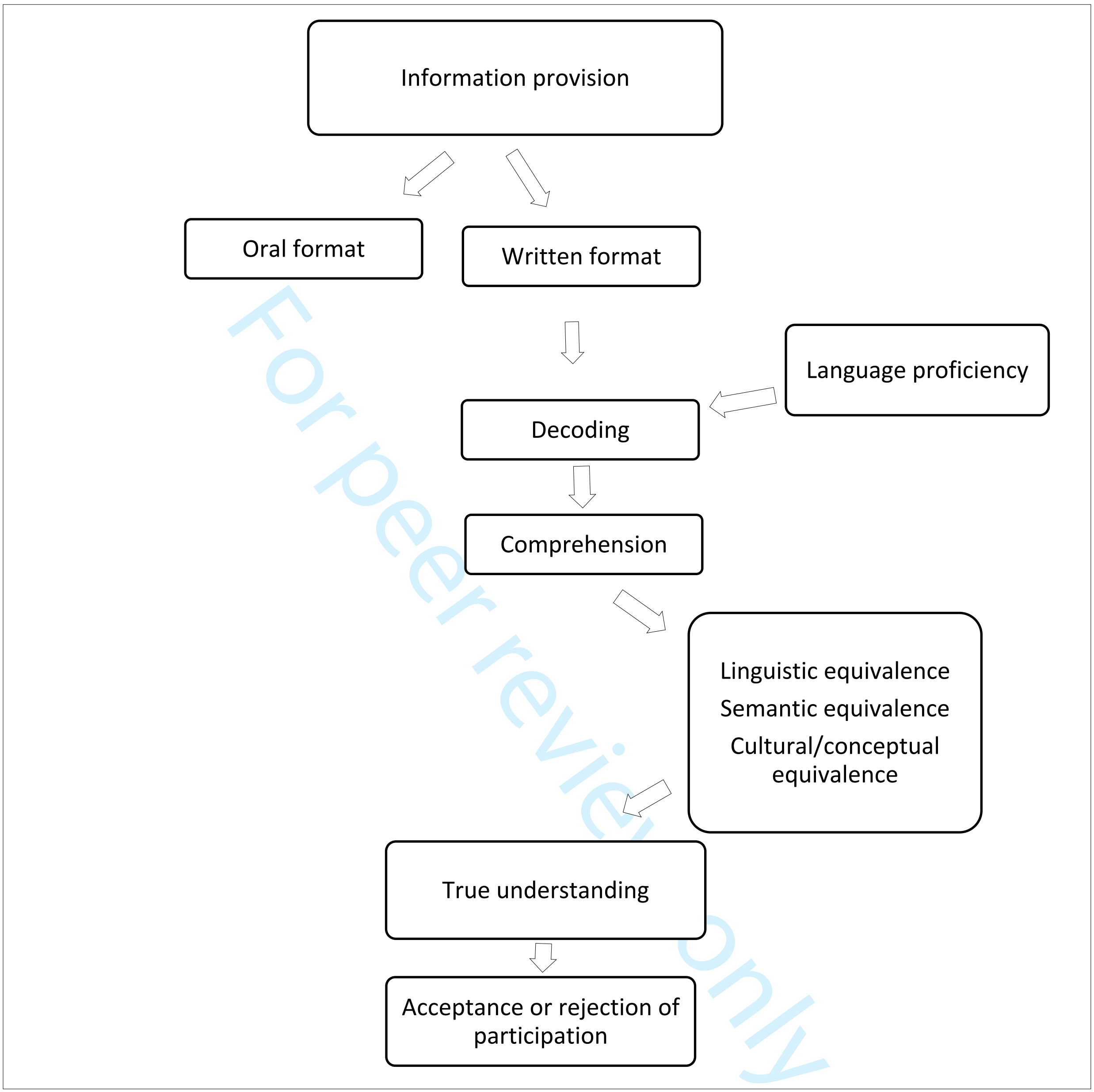

For peer review only

Lost in translation: supplementary material

Contents

Supplementary Table 1: Content analysis of recruitment documents...2

Supplementary Table 2: Interview topic guide...5

Supplementary Table 3: Suggested terms from focus group discussions.......................................7

For peer review only

For peer review only - http://bmjopen.bmj.com/site/about/guidelines.xhtml

Supplementary Table 1: Content analysis of recruitment documents

Theme	Frequency (documents/13)
**Pregnancy specific**	
Pregnancy	6
Pre-eclampsia	5
Delivery	3
Childbirth	3
Placenta	2
Complication	4
Ultrasound	3
Urine sample	3
Umbilical cord	2
Adverse birth outcomes	1
Blood pressure	2
High blood pressure	3
Pregnant women	3
High blood pressure in pregnancy	1
Contractions	1
Planned early delivery	1
Pre-term birth	1
Protein level in the urine	1
**Participation**	
Take part	7
You are being invited to take part in a research project	4
Participation/Participating	10
Withdraw	6
You are free to decline to participate	1
You are free to withdraw from the study at any time	3
You are not forced to participate in this study	2
You do not have to take part in this research if you do not wish to do so	1
Your decision will not affect the affect the care you receive in any way	4
We would like to invite you to take part	1
There is no penalty if you choose not to take part	1
You can receive care whether you take part in our study or not	1
You do not have to be in the research study to receive health care	1

For peer review only

We request your co-operation	1
We would like to invite you to participate in this study	1
**Voluntary Consent**	
Your participation is entirely voluntary	9
Consent	6
Consent form	7
Permission	3
Informed choice	2
I consent voluntarily as a participant in this research	1
If you agree to take part	1
**Risk**	
Concerns	5
Dangerous	2
Risk	8
Serious complications/consequences	5
Side effects	4
Discomfort	5
No risk	4
Suffer	3
Problems	6
Risk factors	1
**Benefit**	
Benefit	6
Benefit others/Benefit to society	2
Benefits to you	1
Direct benefit/may not help you directly/no personal benefit	4
Improve/Improve care/Improve health/outcomes	4
Possible/potential benefits	6
Important benefits	1
**Healthcare care related**	
Your health/Your baby's health	5
Care	4
Symptoms and signs	2
Treatments/Treating	5
Severe problems	2
Samples	3
Results	4
Medicine	3
Injection	4

For peer review only - http://bmjopen.bmj.com/site/about/guidelines.xhtml
For peer review only

Infection	3
Condition	3
Drug/study drug	2
Health	1
Healthcare providers	2
Improve care	2
Swelling	2
Test	3
Swab	2
Health facilities	1
Health information	1
Healthcare	1
**Research concepts**	
What is the purpose of the study	9
Study	11
50/50 Chance	1
Academic collaborators	1
Analysis/Analysed	1
Chance	3
Collect	7
Research	10
Published/publications	3
Knowledge	3
Measure/measurements	3
Presentation at international meetings/conference	2
Sponsor	3
Title of research	1
**Confidentiality/data management**	
Anonymous/Anonymised	4
Confidential	11
Data	5
Kept under lock and key	1
Protected	5
Information	10
Information about you	5
Results	4
Personal information/details	4
Information that is collected about you and your baby	2
Identity	4
Contact information/details	3
Identify	2
Data collected about you	1
Data collection forms	1
Data manager	1

For peer review only - http://bmjopen.bmj.com/site/about/guidelines.xhtml

Supplementary Table 2: Interview topic guide

Details:

**Date**	
**Start time**	
**Finish time**	
**Location**	
**Initial(s) of participant(s)**	
**Age(s)**	
**Level of education**	
**Profession**	

Notes

**Example prompts**<br><br>*What does this term/phrase mean to you?*<br><br>*What do you understand by this term/phrase?*<br><br>*How might this term be translated into Nyanja/Bemba?*<br><br>*Is there an equivalent word?*<br><br>*Is there an equivalent concept?*<br><br>**If participant is a translator:**<br><br>*Can you tell me about your experience of translating research*	

For peer review only

For peer review only - http://bmjopen.bmj.com/site/about/guidelines.xhtml

materials from English to Nyanja/Bemba?<br><br>*Have you come across any challenges?*<br><br>*Is there anything you feel that may improve this process?*<br><br>**If participant is a researcher**:<br><br>*Can you tell me about your experience of using research materials when gaining consent?*<br><br>*Have you come across any challenges?*<br><br>*Is there anything you feel that may improve this process?*	

For peer review only

For peer review only - http://bmjopen.bmj.com/site/about/guidelines.xhtml

Supplementary Table 3: Suggested terms from focus group discussions

English	Nyanja
Pre-eclampsia	Bvuto la kutamanga kwamagazi ndikupezeka kwa dso kudya za mthupi mumitundo pa mene muzimai ali ndi pakati
Stroke	Sitoloko
Low birthweight	Mwana opepoka
Stillborn baby	Nthayo/makanda/ana
Premature baby	Mwana osakosa/osafikapo
Swelling of the legs	Kubvimba kwa mendo
Fitting	Kukunyuka
Urine	Mkhozo/mitundo
Protein in the urine	Kupezeka kwa zakudya zamthupi mu mitundo
High blood pressure	Kutamanga Kwamagazi (Bipi)
This research is brought to you by	Atibweretsera phunziro ndi…/ Akubweretserani…
What are the risks of taking part	Ciyopyezo kuipa/ Kodi kuipa kotengaku mbali ndi kwabwanji?
Research study	Maphunzilo/ Kufunafuna/ Kufufuza
Benefit	Ubwino/Phindu
What are the benefits of taking part?	Ubwino otengako mbali ndiwabwanji?
What is the purpose of the study	Colinga caphunziro ndi ciani?
Why have I been called/invited to take part?	Ndilifukwa ciani/ndaitanidwa kuti ndi tengeko mbali?
What will happen if I take part?	Kodi ndi ciani cizacitika ndi katengaka mbali
Consent form	Cipepala cobvomekeza
Randomisation	Magulu awiri/ Komputa iza zisankila/ Magulu losadzisankhira
Healthy	Umoyo wabwino
Address	Adelesi
Analyse results	Kusanda sanda
Problem/suffering	Mabvuto
The doctor will have to induce labour	Cilikidwa kuyambisidwa

For peer review only - http://bmjopen.bmj.com/site/about/guidelines.xhtml

Standards for Reporting Qualitative Research (SRQR)*

http://www.equator-network.org/reporting-guidelines/srqr/

Title and abstract

	Page/line no(s).
**Title** - Concise description of the nature and topic of the study Identifying the study as qualitative or indicating the approach (e.g., ethnography, grounded theory) or data collection methods (e.g., interview, focus group) is recommended	1
**Abstract** - Summary of key elements of the study using the abstract format of the intended publication; typically includes background, purpose, methods, results, and conclusions	2-3

Introduction

**Problem formulation** - Description and significance of the problem/phenomenon studied; review of relevant theory and empirical work; problem statement	5
**Purpose or research question** - Purpose of the study and specific objectives or questions	6

Methods

**Qualitative approach and research paradigm** - Qualitative approach (e.g., ethnography, grounded theory, case study, phenomenology, narrative research) and guiding theory if appropriate; identifying the research paradigm (e.g., postpositivist, constructivist/ interpretivist) is also recommended; rationale**	9, Figures 1 & 2
**Researcher characteristics and reflexivity** - Researchers' characteristics that may influence the research, including personal attributes, qualifications/experience, relationship with participants, assumptions, and/or presuppositions; potential or actual interaction between researchers' characteristics and the research questions, approach, methods, results, and/or transferability	6-7, 18
**Context** - Setting/site and salient contextual factors; rationale**	6-8
**Sampling strategy** - How and why research participants, documents, or events were selected; criteria for deciding when no further sampling was necessary (e.g., sampling saturation); rationale**	7-8
**Ethical issues pertaining to human subjects** - Documentation of approval by an appropriate ethics review board and participant consent, or explanation for lack thereof; other confidentiality and data security issues	9
**Data collection methods** - Types of data collected; details of data collection procedures including (as appropriate) start and stop dates of data collection and analysis, iterative process, triangulation of sources/methods, and modification of procedures in response to evolving study findings; rationale**	6-8

For peer review only

For peer review only - http://bmjopen.bmj.com/site/about/guidelines.xhtml

**Data collection instruments and technologies** - Description of instruments (e.g., interview guides, questionnaires) and devices (e.g., audio recorders) used for data collection; if/how the instrument(s) changed over the course of the study	7-8, Supplementary Table 2
**Units of study** - Number and relevant characteristics of participants, documents, or events included in the study; level of participation (could be reported in results)	Table 1
**Data processing** - Methods for processing data prior to and during analysis, including transcription, data entry, data management and security, verification of data integrity, data coding, and anonymization/de-identification of excerpts	9-10
**Data analysis** - Process by which inferences, themes, etc., were identified and developed, including the researchers involved in data analysis; usually references a specific paradigm or approach; rationale**	9-10
**Techniques to enhance trustworthiness** - Techniques to enhance trustworthiness and credibility of data analysis (e.g., member checking, audit trail, triangulation); rationale**	10

Results/findings

**Synthesis and interpretation** - Main findings (e.g., interpretations, inferences, and themes); might include development of a theory or model, or integration with prior research or theory	10 - 16
**Links to empirical data** - Evidence (e.g., quotes, field notes, text excerpts, photographs) to substantiate analytic findings	Table 3

Discussion

**Integration with prior work, implications, transferability, and contribution(s) to the field** - Short summary of main findings; explanation of how findings and conclusions connect to, support, elaborate on, or challenge conclusions of earlier scholarship; discussion of scope of application/generalizability; identification of unique contribution(s) to scholarship in a discipline or field	16-18
**Limitations** - Trustworthiness and limitations of findings	18-19

Other

**Conflicts of interest** - Potential sources of influence or perceived influence on study conduct and conclusions; how these were managed	20
**Funding** - Sources of funding and other support; role of funders in data collection, interpretation, and reporting	10, 20

*The authors created the SRQR by searching the literature to identify guidelines, reporting standards, and critical appraisal criteria for qualitative research; reviewing the reference lists of retrieved sources; and contacting experts to gain feedback. The SRQR aims to improve the transparency of all aspects of qualitative research by providing clear standards for reporting qualitative research.

For peer review only - http://bmjopen.bmj.com/site/about/guidelines.xhtml

**The rationale should briefly discuss the justification for choosing that theory, approach, method, or technique rather than other options available, the assumptions and limitations implicit in those choices, and how those choices influence study conclusions and transferability. As appropriate, the rationale for several items might be discussed together.

Reference:

O'Brien BC, Harris IB, Beckman TJ, Reed DA, Cook DA. **Standards for reporting qualitative research: a synthesis of recommendations.** *Academic Medicine*, Vol. 89, No. 9 / Sept 2014 DOI: 10.1097/ACM.0000000000000388

For peer review only

For peer review only - http://bmjopen.bmj.com/site/about/guidelines.xhtml

Understanding the language barriers to translating informed consent documents for maternal heath trials in Zambia: a qualitative study

Journal:	*BMJ Open*
Manuscript ID	bmjopen-2023-076744.R1
Article Type:	Original research
Date Submitted by the Author:	21-Dec-2023
Complete List of Authors:	Beardmore-Gray , Alice ; King's College London, Women and Children's Health<br>Simwinga, Musonda; Zambart<br>Vwalika, Bellington; University Teaching Hospital<br>Chinkoyo, Sebastian; Ndola Teaching Hospital, Department of Obstetrics and Gynaecology<br>Chappell, Dr Lucy; King's College London, Department of Women and Children’s Health, School of Life Course Sciences<br>Sandall, Jane; King's College London, Department of Women and Children's Health<br>Shennan, Andrew; King's College London, Department of Women and Children’s Health, School of Life Course Sciences
**Primary Subject Heading**:	Global health
Secondary Subject Heading:	Ethics
Keywords:	OBSTETRICS, MEDICAL ETHICS, QUALITATIVE RESEARCH

SCHOLARONE™ Manuscripts

I, the Submitting Author has the right to grant and does grant on behalf of all authors of the Work (as defined in the below author licence), an exclusive licence and/or a non-exclusive licence for contributions from authors who are: i) UK Crown employees; ii) where BMJ has agreed a CC-BY licence shall apply, and/or iii) in accordance with the terms applicable for US Federal Government officers or employees acting as part of their official duties; on a worldwide, perpetual, irrevocable, royalty-free basis to BMJ Publishing Group Ltd ("BMJ") its licensees and where the relevant Journal is co-owned by BMJ to the co-owners of the Journal, to publish the Work in this journal and any other BMJ products and to exploit all rights, as set out in our licence.

The Submitting Author accepts and understands that any supply made under these terms is made by BMJ to the Submitting Author unless you are acting as an employee on behalf of your employer or a postgraduate student of an affiliated institution which is paying any applicable article publishing charge ("APC") for Open Access articles. Where the Submitting Author wishes to make the Work available on an Open Access basis (and intends to pay the relevant APC), the terms of reuse of such Open Access shall be governed by a Creative Commons licence – details of these licences and which Creative Commons licence will apply to this Work are set out in our licence referred to above.

Other than as permitted in any relevant BMJ Author's Self Archiving Policies, I confirm this Work has not been accepted for publication elsewhere, is not being considered for publication elsewhere and does not duplicate material already published. I confirm all authors consent to publication of this Work and authorise the granting of this licence.

For peer review only

Understanding the language barriers to translating informed consent documents for maternal heath trials in Zambia: a qualitative study

Alice Beardmore-Gray[a], Musonda Simwinga[b], Bellington Vwalika[c], Sebastian Chinkoyo[d], Lucy C Chappell[a], Jane Sandall[a]*, Andrew H Shennan[a]*.

Author affiliations

[a]Department of Women and Children's Health, School of Life Course Sciences, King's College London, London, UK (alice.1.beardmore-gray@kcl.ac.uk, lucy.chappell@kcl.ac.uk, jane.sandall@kcl.ac.uk, andrew.shennan@kcl.ac.uk)

[b]Zambart, Lusaka, Zambia (musonda@zambart.org.zm)

[c]Department of Obstetrics and Gynaecology, University of Zambia, Lusaka, Zambia (vwalikab@gmail.com)

[d]Department of Obstetrics and Gynaecology, Ndola Teaching Hospital, Ndola, Zambia (seb.chinkoyo@yahoo.com)

Correspondence: alice.1.beardmore-gray@kcl.ac.uk

*JS and AS are joint senior authors.

For peer review only

Abstract

Objective

Providing comprehensible information is essential to the process of valid informed consent. Recruitment materials designed by sponsoring institutions in English-speaking, high-income countries are commonly translated for use in global health studies in other countries; however, key concepts are often missed, misunderstood or "lost in translation". The aim of this study was to explore the language barriers to informed consent, focusing on the challenges of translating recruitment materials for maternal health studies into Zambian languages.

Design

We used a qualitative approach, which incorporated a multi-stakeholder workshop (11 participants), in-depth interviews with researchers and translators (8 participants), and two community-based focus groups with volunteers from community advisory boards (20 participants). Content analysis was used to identify terms commonly occurring in recruitment materials prior to the workshop. The framework analysis approach was used to analyse interview data, and a simple inductive thematic analysis approach used to analysis focus group data.

Setting

The study was based in Lusaka, Zambia.

Results

The workshop highlighted difficulties in translating research terms and pregnancy-specific terms, as well as widespread concern that current templates are too long, use overly formal language, and are designed with little input from local teams. Framework analysis of in-depth interviews identified barriers to participant understanding relating to design and development of recruitment materials,

For peer review only - http://bmjopen.bmj.com/site/about/guidelines.xhtml

language, local context, and communication styles. Focus group participants confirmed these findings and suggested potential solutions to ensure the language and content of recruitment materials can be better understood.

Conclusion

Our findings demonstrate that the way in which recruitment materials are currently designed, translated, and disseminated, may not enable potential trial participants to fully understand the information provided. Instead of using overly complex institutional templates, recruitment materials should be created through an iterative and interactive process that provides truly comprehensible information in a format appropriate for its intended participants.

For peer review only - http://bmjopen.bmj.com/site/about/guidelines.xhtml

Strengths and limitations

- The use of a mix of qualitative data collection methods (interviews and focus groups) triangulated and enhanced the reliability of our findings, and ensured representation of a broad range of perspectives.
- Inclusion of additional stakeholders, such as members of ethical review boards, could have provided more information on the issue of informed consent, particularly regarding the ethical review process for recruitment materials.
- The inclusion of community advisory board members strengthened our study by providing an important community voice; however, inclusion of a wider range of individuals from the community, including those more likely to be marginalised, and pregnant women in particular, could have ensured wider representation and added further to our findings.
- The challenges described in this study are likely to be country and context specific. Our findings may inform other maternal health researchers working in Zambia, as well as outlining important principles which may apply to similar settings.

For peer review only

For peer review only - http://bmjopen.bmj.com/site/about/guidelines.xhtml

Introduction

Statement of the problem

Global health research typically involves partnerships between high income and low or middle income countries. These partnerships can sometimes perpetrate inherent structural inequalities or power dynamics,(1-5) whereby research methodology and institutional processes designed in a high income country may be imposed on low income partners without considering the relevance or acceptability to the local population. The process of informed consent, and ethical review of consent documents, are two of the domains which may be affected by this imbalance. This study evaluates an example within the context of a maternal health trial conducted in Lusaka, Zambia, specifically exploring how language barriers, and issues surrounding translation of recruitment materials, may impact upon informed consent.

Informed consent is fundamental to any research involving human beings. For consent to be valid, participants must have the capacity to consent, act voluntarily, and be provided with sufficient comprehensible information. These principles are well described and upheld by international ethical and legal frameworks.(6, 7) However, these frameworks are based upon knowledge systems generated and perpetuated by dominant groups in high-income countries(1) and often imposed upon other communities without considering local expertise. The participant information leaflets and consent forms (recruitment materials) used for enrolling participants into clinical trials conducted in low or middle income countries are often designed by sponsoring institutions based in high-income countries(8) and are therefore more likely to meet the needs of trial sponsors and ethical review boards, rather than those of the intended participants. There is a focus on written documentation, complex medico-legal language and lengthy forms providing excessive information. These forms are then translated via a process of forward and back translation, into the local language(s) of the country where the research is taking place. However, a 2014 review into

For peer review only - http://bmjopen.bmj.com/site/about/guidelines.xhtml

participant comprehension found that the majority of trial participants across different African countries did not understand several key domains of informed consent such as voluntariness, confidentiality, and the difference between taking part in research and seeking medical care.(9) This is attributed to a lack of conceptual equivalence,(10-13) arising from a lack of directly equivalent terms, as well as languages that are predominantly spoken and therefore do not have standardised written formats. Use of overly complex words and medical terminology further exacerbates this lack of understanding.(14) Studies have also highlighted a lack of universal tools for assessing understanding of trial participants(9, 15-17) and this in itself presents a barrier to identifying areas for improvement. Several studies have highlighted linguistic factors as a significant barrier to comprehension, but there is very little literature exploring this particular issue. Maternal health is a key research priority which justifiably attracts large numbers of research studies. However, pregnant women are a vulnerable population and in many low or middle income countries, including Zambia, vulnerability may be compounded by low levels of educational attainment and literacy.(18) By exploring the language barriers to cross-cultural adaptation of recruitment materials for a maternal health-related clinical trial, we aim to improve the quality of recruitment materials provided to future participants in maternal health studies in Zambia, and to contribute towards local efforts to strengthen research ethics capacity, which has been identified as a key priority by the Zambia National Health Research Policy and the Zambian National Health Regulatory Authority.(19)

Research objective

The overall aim of this study is to understand the language barriers to informed consent, and to demonstrate, via the example of translating maternal health research materials in Zambia, the importance of developing informed consent processes and providing participant information in a way that truly suits the needs of research participants.

For peer review only - http://bmjopen.bmj.com/site/about/guidelines.xhtml

Methods

We used a qualitative study design incorporating a participatory workshop, in-depth interviews, and focus group discussions. This study took place in three phases (Table 1), based primarily in Lusaka, Zambia, alongside a timing of delivery in pre-eclampsia trial;(20) the research was led by the coordinator of this trial, a UK doctor. An SRQR checklist is provided in the supplementary materials.

For peer review only

For peer review only - http://bmjopen.bmj.com/site/about/guidelines.xhtml

Table 1: study phases and participants

Phase	Activity	Participant summary
Phase 1 Lusaka 18th Nov 2019	Facilitated workshop with invited participants from a variety of professional backgrounds. We set out to explore how key maternal health research terms, identified from a cross-sectional sample of recruitment materials, from different research studies, might be translated from English into Nyanja and Bemba and how this process might alter their meaning, as part of an initial exploratory exercise to guide the subsequent two phases	There were 11 participants including ABG (study lead). Five participants were female, and six were male. Nine were Zambian, two were British. Four were Obstetric researchers, three were research assistants and four were translators with a background in teaching and social science. Participants were invited based on their ongoing involvement with a clinical trial evaluating timing of delivery in pre-eclampsia
Phase 2 13th May – 1st July 2021	In depth interviews with key informants to understand in more detail the challenges involved with translating consent documents for a Zambian population	A total of eight interviews took place. The age range of participants was 30 to 69, three were female and five were male. Most (six) had degrees, two had diplomas. Their occupations included language teacher (three participants), research coordinator (four participants) and one community engagement officer
Phase 3 21st and 29th June 2021	Focus group discussions with local community advisory board members at primary health clinics to interrogate findings from Phases 1 and 2 with individuals who would be representative of potential research participants	A total of two focus group discussions (20 participants in total, ten in each group) took place. The mean age of participants was 28 years, twelve were female and six were male (information not provided for two participants). Eight participants had attended tertiary level education, with the remainder having attended secondary level education

For peer review only

Sampling strategy and data collection methods

The cross-sectional sample of recruitment materials used during Phase One was obtained by inviting researchers working in Zambia to submit English language examples of recruitment materials they had previously developed (and subsequently translated) to inform individuals considering participation in their research studies (predominantly clinical trials). Researchers were identified via ongoing research being conducted at University Teaching Hospital, Lusaka, ongoing research conducted by the Department of Women and Children's Health at King's College London, and via the Global Women's Research Society international conference. Researchers were asked to provide English language versions of participant information leaflets which were collated, read, and analysed

For peer review only - http://bmjopen.bmj.com/site/about/guidelines.xhtml

by the study lead (ABG). All relevant examples of recruitment materials provided were included in the total sample of 13 documents. Summative content analysis (see Data analysis section) was used to identify the most commonly occurring terms related to research and pregnancy (details shown in Supplementary Table 1). These terms were organised into relevant themes such as pregnancy-specific terms, research concepts and confidentiality (Supplementary Table 1). The workshop focussed on how these different English terms could be translated for a Zambian population, and the potential difficulties that might be encountered when doing so. Through our discussion with workshop participants, we were able to identify which commonly occurring terms were most difficult to translate. Contemporaneous group notes were made on flip charts during this process. In addition, the independently performed back translations of participant information leaflets (translated as part of the timing of delivery in pre-eclampsia trial conducted in Zambia) were also discussed. These leaflets had been translated from English, into Nyanja, and then back to English. The discussion focussed on comparing and contrasting the original English versions with the back-translated versions.

Phase Two in-depth interviews with key informants were significantly delayed due to the Covid-19 pandemic. An initial convenience sample of key informants was used, whereby individuals were invited to participate if they had prior experience of either translating recruitment materials or enrolling participants into research studies. As data collection continued, additional informants were invited to participate via snowball sampling. This comprised inviting other individuals, suggested by key informants, who were likely to have relevant insight and expertise, such as community engagement officers or research assistants. Interviews were conducted in English, the working language in Zambia, by the study lead (ABG). A semi-structured interview guide was used (Supplementary Table 2) and each interview was audio recorded and then transcribed. The interviews took place at times and locations convenient to the participants, primarily office spaces and meeting venues in Lusaka, Zambia. Phase Three focus group discussions with community advisory board members were facilitated by three of the professional language teachers/translators

For peer review only

For peer review only - http://bmjopen.bmj.com/site/about/guidelines.xhtml

who had participated in the in-depth interviews (phase 2) as key informants, supported by the study lead (ABG). Focus group participants were invited by asking community advisory board members to participate if they wished. Community advisory board members were volunteers from the local community who were part of pre-existing community groups, linked to primary healthcare facilities in Lusaka, Zambia. Community advisory board members linked to Kanyama first level hospital and Chawama first level hospital were selected as these are two of the busiest primary healthcare facilities in Lusaka and both facilities had enrolled participants into the previously mentioned timing of delivery in pre-eclampsia trial. Invitations were sent out to community advisory board members via text message, and responding individuals were then invited to participate in a focus group discussion. Two initial focus groups were planned, as a purposeful sample, designed to interrogate findings from the workshop and key informant interviews. A focus group guide was developed following the Phase Two interviews and adapted from the interview topic guide (Supplementary Table 2). Focus group discussions took place in outdoor meeting spaces attached to two first level hospitals (Kanyama and Chawama) in Lusaka, and were audio recorded and transcribed. Focus groups were conducted in a mixture of English, Nyanja and Bemba and were translated at the time of transcription by a Zambian research assistant.

Ethical considerations

Ethical approval for this study was provided by King's College London (MRSP-20/21-22350) and University of Zambia Biomedical Research Ethics Committee (1517-2020). Written informed consent was sought from all participants before any interviews or focus group discussions were conducted and participation in the study was entirely voluntary. Electronic copies of interview and focus group transcripts were stored on a password protected hard drive. Participants were anonymised and referred to by initials or numbers only.

For peer review only - http://bmjopen.bmj.com/site/about/guidelines.xhtml

Data analysis

Content analysis was used to analyse recruitment materials as a recognised method of rapidly identifying commonly occurring language. A summative approach was taken, identifying the number of times that pregnancy related phrases and research terms arose in the sample of recruitment materials.(21) During the Phase One workshop (conducted in English), participants discussed the interpretation of these commonly occurring English terms, and explored how they might be translated into Nyanja and Bemba. Key outputs from the discussion notes were used to inform the design of Phases Two and Three. Interview transcripts from Phase Two were uploaded to NVivo 12 for data coding. A framework analysis approach was used to analyse interview data.(22) The theory underpinning this framework was drawn from the conceptual framework for the process of obtaining informed consent outlined by Bhutta,(8) theories of reading and language proficiency,(23) and models for translation and cross-cultural adaptation such as those outlined by Brislin(24) and Flaherty et al.(25) These theories were combined into one overarching framework (Supplementary Figure 1(14, 18, 24, 25)) which guided data analysis, and was developed further during the analysis process, informing the final thematic framework shown in Figure 1. Focus group data were analysed using a simple inductive thematic analysis approach. The themes identified were compared and contrasted to findings from the interview data. By collecting data using different methods (workshop, interviews, and focus group discussions) and from different sources (e.g. research professionals and community members) we were able to triangulate our data (26) and test the validity of our findings from each phase, thereby enhancing the trustworthiness of our data. Focus group discussions with community members were chosen as a method of interrogating the findings from the workshop and interviews, and to seek differing perspectives and suggestions from individuals likely to represent potential research participants (as part of a local community linked to primary healthcare facilities involved in recruiting to clinical trials).

For peer review only - http://bmjopen.bmj.com/site/about/guidelines.xhtml

Role of the funding source

The funder of the study had no role in study design, data collection, data analysis, data interpretation, or writing of the report. The corresponding author had full access to all the data in the study and had final responsibility for the decision to submit for publication.

Patient and public involvement

Patients and the public were not involved in the design or implementation of this study. However, the initial phase, a multi-disciplinary workshop, incorporated individuals from a range of professional backgrounds including social scientists and language teachers in addition to clinicians and researchers. Subsequent phases involved professional translators and members of the public in the form of community advisory boards, with the overall findings and recommended actions reflecting their lived experience and perspectives.

Results

Phase One: initial workshop

During the Phase One workshop, participating translators highlighted the lack of equivalent terms (in Nyanja or Bemba) for words such as “pre-eclampsia”, “proteinuria”, and “contractions”, as well as differing interpretations of words such as “research”, “benefits” and “risks”. The word “consent” itself was also raised as a term which could be interpreted differently depending on the context, with some communities being less familiar with the concept of individualised consent than others. Important examples of discrepancies identified during discussion of the back translations are shown in Table 2. This interactive workshop highlighted several concerns regarding current procedures for designing and translating research documents, and the lived experiences of the participants suggested this was a common and widespread issue. The group therefore proposed further exploration of the language barriers to adaptation of maternal health recruitment materials in

For peer review only

For peer review only - http://bmjopen.bmj.com/site/about/guidelines.xhtml

220 Zambia via in-depth interviews, followed by community focus group discussions to develop locally
221 driven solutions that may be generalisable to other researchers working in similar settings.

222 **Table 2: Examples of back translations**

Original (English)	*Back translation (from Nyanja translation)*
We appreciate your time and are grateful for your help. However, there will not be any financial compensation for taking part in this study. By choosing to take part in this study you will be helping us to help other women like you in the future.	We are very thankful for giving us your time and all the help that you have rendered to us. Even if things are like this, they will be no funerals of any kind because you have taken part in this research study.
If you take part in the study we will collect some personal information. This will only be used by members of the research team if they need to contact you. This information will be kept confidential. This means that only members of the research team will have access to it, and it will not be shared with anybody else. The data will be protected according to UK Data Protection Laws.	I agree that my suggestions that I will give should be made use of in this lesson (the way my suggestions have been presented) I am aware that my suggestions will be kept following the best recommended practices of keeping secrets.
CRADLE-4 Phase 1: The feasibility and acceptability of planned early delivery in pre-eclampsia in a Low and Middle-Income Setting.	Reviewing the advantages of early childhood delivery on the poor women and those at the centre of fending for their families. This is usually centered on the women with complications of swelling of feet and other body parts, excess proteins in the blood and urine and high blood pressure.
You have been invited to take part in this study because you have pre-eclampsia, but your condition does not require that your baby be delivered immediately.	Therefore, you are requested to take part in this study so as to help us get the facts regarding this matter.

223

224 **Phases Two and Three: In-depth interviews and focus group discussions**

225 The initial theoretical framework was modified throughout the coding process, with the final
226 thematic framework used for data analysis shown in Figure 1.

For peer review only - http://bmjopen.bmj.com/site/about/guidelines.xhtml

Design and Development of recruitment materials

The interview participants working as research coordinators felt they were not given sufficient opportunity to contribute to the design of recruitment materials at an early stage, stating that they are often invited to review documents only after they have already been finalised and submitted to the ethics committee (Table 3, quote D1). Information leaflets were criticised as being too long and wordy, with emphasis placed on the need to present key messages more succinctly using alternative methods such as flyers, community announcements and household visits. The translation process itself was identified as a significant issue, due to an over-emphasis on literal word for word translations, rather than communicating the true meaning of the information. This issue was felt to be exacerbated by poor interactions between researchers and translators. The professional translators interviewed spoke of pressure to produce work within a tight timeframe, compounded by a lack of face-to-face meetings with researchers meaning that research principles and scientific concepts were often not thoroughly understood by the individual translating the document (Table 3, quote D2). Language itself was an important barrier, primarily due to a lack of equivalence – often there is simply no equivalent word in the local language for a particular English medical term. As a result, translators may try to explain the term using multiple words and phrases which ultimately distort or change the meaning (Table 3, quote D3). Furthermore, a clear distinction was made between "play" language and "formal" language with some translators criticising the overuse of formal language in translated documents, rendering them incomprehensible to the intended recipients who use different, more colloquial versions. Finally, the presence of multiple languages in Zambia (72 in total) was identified as a further challenge, as most documents will be translated into just a few of these languages which will be understood to varying degrees by different individuals depending on their family background and where they live.

Focus group participants also felt that information in recruitment documents should be shortened and simplified and that lengthy information relating to the sponsoring institution and data

For peer review only - http://bmjopen.bmj.com/site/about/guidelines.xhtml

protection was not necessary. Many participants felt that verbal explanations, audio-visual aids and flip charts could enhance information provision but agreed with interview participants that written documentation was an important component of the process that should not be eliminated. Participants felt that greater emphasis needed to be placed on the voluntary nature of study participation, with statements such as “you do not have to take part if you do not want to” given greater prominence and translated clearly and directly to ensure the meaning was clear. Participants also stated that language related to funding needed to be clarified, as direct translation of the English phrasing implied possible financial incentives could be provided by taking part. Participants also provided examples of different terms that may be used to explain pregnancy or birth depending on the context, and that whilst informal terms were sometimes considered less “respectful”, they were often better understood by their community (Table 3, quote D4). There was tension between some translators who wished to preserve the formal, grammatically correct version of their language as taught in schools and focus group participants who preferred more colloquial terms. A suggested solution was using more informal terms in brackets so that both the official and colloquial terms could be presented and communicated effectively, depending on the user. Focus group participants also suggested creating a glossary of certain words at the start of any document, using local terms to explain in detail medical terms such as pre-eclampsia or proteinuria for the reader. Participants expressed specific preferences for different translations of particular words, examples of which are presented in Supplementary Table 3. Throughout, more informal versions were preferred, and alternative terms suggested which were sometimes different from the versions originally provided by translators.

For peer review only

Table 3: Illustrative quotes

**Design and development**	
D1.“what I noticed is that we just receive the consent, you can’t change anything in the consent”	Interview participant

For peer review only - http://bmjopen.bmj.com/site/about/guidelines.xhtml

D2. "There are people, some people, would have sent work, you work on their consignment, you just send back. You've never met face to face. They have no time to sit with you"	Interview participant
D3. "pre-eclampsia in our local language, we don't have it, it's not there, so a translator need to have a rich vocabulary and full understanding for you to come up with the correct translation"	Interview participant
D4. "here in Lusaka they don't use kubeleka, but instead they say (abala) so for this word, it will be difficult for the community to understand"	Focus group participant
**Context and communication**	
C1. "you will find that some people, when they find these women who maybe can't read on their own and they have to read for them, so you will find that most of the time, there is this issue of inadequate information being given and it will be like fast done"	Interview participant
C2. "you need to get consent from the husband and yet the pregnant woman is an adult, so they can consent on their own but they will not consent, they want consent from their husband or from their parents"	Interview participant
C3. "people need to understand, what is ultrasound, what is this machine, why are you doing this on me? What is its effect"	Interview participant
C4. "HIV, where you are doing blood draws so they would, from communities, they would, they would think you are selling their blood"	Interview participant
C5. "looking at the community where we come from, the people that read this information trust me, most of them can't read, most of them can't even read the local language"	Focus group participant

Context and communication

The way in which information is communicated to participants, as well as the context into which it is being delivered, was highlighted by both interview and focus group participants as an important area needing improvement. Some interview participants felt that potential participants are not given sufficient time to consider the information provided, with decisions often expected on the same day that a study is explained for the first time by research teams. Furthermore, some researchers described often needing to verbally explain recruitment materials to illiterate participants. They felt that this makes it difficult to standardise the information provided to potential participants and risks potential participants receiving insufficient or even inaccurate information (Table 3, quote C1). When considering the context into which translated documents are being introduced, all of the

For peer review only

For peer review only - http://bmjopen.bmj.com/site/about/guidelines.xhtml

interview participants raised the importance of the target population and the need to consider the levels of literacy, the languages used, the age and gender of potential participants (for example many pregnant women require their husband's consent before participating in any study) and also the common misconceptions that may be prevalent within that community surrounding healthcare interventions or research studies (Table 3, quotes C2 to C4). Many interview participants highlighted the fact that use of inappropriate language or poorly designed forms will compound this issue, and risks both limiting the number of potential participants enrolled into a study and undermining the validity of the informed consent of those who do decide to take part.

Focus group participants raised similar concerns, recalling having previously been given brochures or leaflets to read, and not having the time or inclination to do so. Having more in-depth discussions, with audio-visual aids, and the opportunity for further discussions to ask questions at a later date were suggested as measures that may improve participant comprehension. Consistent with interview findings, focus group participants highlighted the importance of understanding the target community and in particular mentioned the fact that, in their experience, most individuals in their community could not read the local language (Table 3, quote C5). They felt that simple information should be provided in ways that are easy to understand such as flip charts and pictures. However, the background and education of potential participants was also highlighted as an important factor to consider when choosing the most appropriate information format – with participants suggesting that in some communities, video consent may be deemed suspicious or inappropriate. Geographical region was also highlighted as important, with preferred terms changing depending on which area of the country the research is being conducted.

Discussion

Our collaborative workshop highlighted the discrepancies between the original English versions of recruitment materials and translated copies, as well as the difficulty in finding equivalent terms to accurately convey the intended meaning of key research concepts and medical words such as "pre-

For peer review only - http://bmjopen.bmj.com/site/about/guidelines.xhtml

eclampsia". We identified several barriers to participant comprehension and informed consent within in-depth interview data, including a lack of time available to translators, poor literacy, and rushed interactions between researchers and potential participants. Researchers working in Zambia felt that the content and layout of recruitment materials were designed by "the owners" in English speaking countries and that they had little opportunity to influence the design or make their voices heard, with translations subsequently regarded as poor quality. In contrast to the grammatically correct, formal translations often used by professional translators, focus group participants expressed a clear preference for translated versions of recruitment materials to use more informal language, and that this should vary depending on the target population of a study. Furthermore, whilst workshop participants suggested audio-visual aids as a potential solution, interview and focus group participants felt that although they may be a helpful supplement, it was important to have hard copies of written information to refer back to and maintain trust.

Previous research on informed consent has focused primarily on identifying gaps in participants' understanding and evaluating community perceptions of research. Our findings correlate with those described by other studies, which found that there were widespread misconceptions regarding the purpose of research, the benefits and risks of taking part, and the use of research samples such as blood samples.(27, 28) If the content of research documents does not address peoples' fears and beliefs (for example around blood tests or ultrasound scans) and explain in detail what is expected of participants and why, participants may base their decision on whether to participate or not on misinformation. Previous studies highlighted a need to further investigate the language barriers to effective communication about research, as well as to develop pre-tested and standardised tools that can be used to explain research concepts in a way the local community can understand. However, ours is the first study to our knowledge which explores these barriers, with a focus on translating recruitment materials and a specific focus on maternal health terms. We therefore build upon the issues raised by previous work, exploring the specific difficulties relating to language and conceptual equivalence in more detail, adding voices from a cross-section of individuals in Zambia,

For peer review only

For peer review only - http://bmjopen.bmj.com/site/about/guidelines.xhtml

directly involved in the design and implementation of maternal health research, as well as community representatives of target populations.

There has been a call to action within the global health community to redress the systemic imbalances that are perpetuated by Eurocentric institutions and practices.(29) However, there are very few worked examples that demonstrate how these inequities may cause harm to research participants, and even fewer examples that suggest ways of dismantling these practices.(30) This study provides a practical and tangible example of ways in which researchers and ethical review boards can begin the process of change right away. A recent scoping review highlighted the financial, administrative and regulatory barriers to good quality ethical review in low and middle-income countries;(31)our study provides relevant findings that may be used to address some of these concerns. A collaborative, multidisciplinary research programme in Kenya has successfully implemented a systematic approach to translating contextualised informed consent templates, drawing on community engagement processes within their research programme, which has received positive engagement from researchers and ethics committees.(32) We present our own summary of our recommended actions for institutions, researchers, and translators in Figure 2, which represents the perspectives of the Zambian participants in this study, and could be used to inform a similar approach in a Zambian setting.

Strengths and Limitations

The initial research question and subsequent study design were influenced by the experiences of the study coordinator (ABG) when translating recruitment materials for the feasibility study informing the main timing of delivery in pre-eclampsia trial,(20, 33) which suggested the specific difficulties encountered during this process may represent a wider issue. This was explored further during the course of data collection and analysis, acknowledging the potential biases that may have been carried forwards from this initial experience. Collecting data from different sources helped to counteract any inherent individual bias. For example, the assumption that participants might prefer

For peer review only - http://bmjopen.bmj.com/site/about/guidelines.xhtml

information provided in alternative formats was dispelled by both interview and focus group participants who felt it was important to have a written, hard copy of any recruitment materials. In their position as a trial coordinator, it is possible that interview and focus group participants may have viewed them as possessing a certain level of authority, and this in turn may have influenced the responses of the participants. Steps taken to counteract this included a relaxed communication style during interviews and using local translators to help facilitate focus group discussions. In their position as a researcher based in Zambia during the time period that this study took place, the study lead was able to connect with and seek out key informants within the local research community and seek guidance from local experts working in social science research. Language teachers and translators represented an important group of participants for this research. Whilst they had previous professional experiences of translating research materials, it was clear that the objective of this study was to learn from and understand their experiences, rather than engage them in a professional capacity, thus limiting the potential for any conflict of interest. The translators who performed the initial translations used for the back-translations discussed at the workshop each worked as professional teachers of either Nyanja, Bemba, or both, in the public education system in Zambia. Each translator had at least five years' experience of translating research documents for clinical trials. Although some of the discrepancies identified may be related to errors, rather than specific language barriers (for example, the addition of the word "funerals" in the first example provided in Table 2), this highlights the importance of performing back translation (not always required by ethical review bodies), and allowing sufficient time for translators and researchers to meet face to face and discuss their work, an important process which, according to the translators interviewed, was frequently ignored by researchers. Focus group participants were recruited from local community advisory boards. These groups are local volunteers who are often consulted to gain community input and perspectives on healthcare interventions and research studies. Whilst this meant they were well-placed to participate in the focus group discussions facilitated as part of this

For peer review only - http://bmjopen.bmj.com/site/about/guidelines.xhtml

study, participants outside of this well-established model may have provided a wider array of insights.

The views of both interview and focus group participants likely represent an urban population, though many interview participants had experience of a wide range of research studies conducted over different time periods and in different areas of the country. Interview and focus group participants had many experiences of research, given that they lived in Lusaka, the capital city, where many of the healthcare facilities have ongoing involvement in several research projects. A more remote setting in areas where participants are less familiar with research may have provided different findings. However, given the aim of this study was to specifically explore issues when translating, using, and understanding participant information documents, the selected population was likely appropriate for the research objectives. Inclusion of ethical review board members, or study principal investigators, who are responsible for approving many of the recruitment materials used in global health studies, could have added an additional and important perspective on the issues explored in our study. Engaging these key stakeholders would be important in any future research, and when implementing our recommended actions.

In this study, we focus on the example of translating recruitment materials for a maternal health-related clinical trial in Zambia. It is possible, that the specific challenges described by participants in this study, may not necessarily apply to other study designs or contexts, thereby limiting the generalisability of our findings. However, by sharing the key learning points identified from this qualitative study, it may prompt any individual involved in translating or using participant information in similar settings to critically review the language they use, and whether it is appropriate and comprehensible to its intended audience.

Conclusions

Our study has identified that current methods of designing and translating recruitment materials for potential research participants in maternal health studies in Zambia, may not always facilitate true

For peer review only - http://bmjopen.bmj.com/site/about/guidelines.xhtml

understanding, and therefore may not serve the needs of their intended recipients. This problem requires researchers and ethics committees to re-evaluate their current practice and move away from viewing translation as merely a tick-box exercise required to gain ethical approval, but a collaborative and dynamic process that can be adapted to suit the needs of the communities, countries, and languages in which the research is taking place.

Declaration of interests

The authors declare no conflicts of interest.

Author Contributions

ABG, AS and LCC were involved in the study conception and in securing funding for the study. ABG, MS, BV, SC, LCC, JS and AS designed the study protocol and secured ethical approval for the study. ABG coordinated the study supported by BV and SC. ABG did the study analysis with input from MS and JS. ABG wrote the original manuscript draft. All authors reviewed, contributed to, and approved the final version of the manuscript.

Funding

This study was funded by the King's ODA Research Partnership Seed Fund (KODA_1819_002). The funder of the study had no role in study design, data collection, data analysis, data interpretation, or writing of the report. The corresponding author had full access to all the data in the study and had final responsibility for the decision to submit for publication.

Acknowledgements

We thank all the individuals who participated this study, providing their valuable time and insight. Jane Sandall is an NIHR Senior Investigator and is supported by the National Institute for Health Research (NIHR) Applied Research Collaboration South London (NIHR ARC South London) at King's

For peer review only - http://bmjopen.bmj.com/site/about/guidelines.xhtml

College Hospital NHS Foundation Trust. The views expressed are those of the author[s] and not necessarily those of the NIHR or the Department of Health and Social Care.

Data sharing

The dataset will be available to appropriate academic parties on request from the corresponding author in accordance with the data sharing policies of King's College London, with input from the co-author group where applicable.

For peer review only

For peer review only - http://bmjopen.bmj.com/site/about/guidelines.xhtml

References

1. Bhakuni H, Abimbola S. Epistemic injustice in academic global health. Lancet Glob Health. 2021;9(10):e1465-e70.
2. Zarowsky C. Global health research, partnership, and equity: no more business-as-usual. BMC Int Health Hum Rights. 2011;11 Suppl 2(Suppl 2):S1.
3. Rasheed MA. Navigating the violent process of decolonisation in global health research: a guideline. Lancet Glob Health. 2021;9(12):e1640-e1.
4. Horton R. Offline: Challenging America's hegemony in global health. The Lancet. 2013;382(9890):382-.
5. Hommes F, Monzo HB, Ferrand RA, Harris M, Hirsch LA, Besson EK, et al. The words we choose matter: recognising the importance of language in decolonising global health. Lancet Glob Health. 2021;9(7):e897-e8.
6. World Medical Association. WMA Declaration of Helskini - Ethicial Principles for Scientific Requirements and Research Protocols 2013 [29-32]. Available from: https://www.wma.net/policies-post/wma-declaration-of-helsinki-ethical-principles-for-medical-research-involving-human-subjects/.
7. Council for International Organizations of Medical Sciences. International Ethical Guidelines for Health-related Research Involving Humans Fourth Edition. Geneva. 2016. p. 1-122.
8. Bhutta ZA. Beyond informed consent. Bull World Health Organ. 2004;82(10):771-7.
9. Afolabi MO, Okebe JU, McGrath N, Larson HJ, Bojang K, Chandramohan D. Informed consent comprehension in African research settings. Trop Med Int Health. 2014;19(6):625-42.
10. Oduro AR, Aborigo RA, Amugsi D, Anto F, Anyorigiya T, Atuguba F, et al. Understanding and retention of the informed consent process among parents in rural northern Ghana. BMC Med Ethics. 2008;9:12.
11. Marshall PA. "Cultural competence" and informed consent in international health research. Camb Q Healthc Ethics. 2008;17(2):206-15.
12. Gjersing L, Caplehorn JR, Clausen T. Cross-cultural adaptation of research instruments: language, setting, time and statistical considerations. BMC Med Res Methodol. 2010;10:13.
13. Tekola F, Bull SJ, Farsides B, Newport MJ, Adeyemo A, Rotimi CN, et al. Tailoring consent to context: designing an appropriate consent process for a biomedical study in a low income setting. PLoS Negl Trop Dis. 2009;3(7):e482.
14. Villafranca A, Kereliuk S, Hamlin C, Johnson A, Jacobsohn E. The Appropriateness of Language Found in Research Consent Form Templates: A Computational Linguistic Analysis. PLoS One. 2017;12(2):e0169143.
15. Appelbaum PS. Understanding "Understanding": An Important Step Toward Improving Informed Consent to Research. AJOB Primary Research. 2010;1(2):1-3.
16. Sand K, Kaasa S, Loge JH. The Understanding of Informed Consent Information—Definitions and Measurements in Empirical Studies. AJOB Primary Research. 2010;1(2):4-24.
17. Lindegger G, Milford C, Slack C, Quayle M, Xaba X, Vardas E. Beyond the checklist: assessing understanding for HIV vaccine trial participation in South Africa. J Acquir Immune Defic Syndr. 2006;43(5):560-6.
18. Zambia Statistics Agency, Ministry of Health Zambia, ICF International. Zambia Demographic and Health Survey 2018. Lusaka, Zambia and Rockville, Maryland, USA: Zambia Statistics Agency, Ministry of Health, and ICF.; 2019.
19. National Health Research Authority. Building capacity for research ethics and regulation in Zambia 2020 [https://www.nhra.org.zm/new-https://www.nhra.org.zm/new]. Available from: https://www.nhra.org.zm/new-https://www.nhra.org.zm/new
20. Beardmore-Gray A, Vousden N, Charantimath U, Katageri G, Bellad M, Kapembwa K, et al. Planned early delivery versus expectant management to reduce adverse pregnancy outcomes in pre-eclampsia in a low- and middle-income setting: study protocol for a randomised controlled trial (CRADLE-4 Trial). Trials. 2020;21(1):960.

For peer review only - http://bmjopen.bmj.com/site/about/guidelines.xhtml

21. Hsieh H-F, Shannon SE. Three approaches to qualitative content analysis. Qualitative health research. 2005;15(9):1277-88.

22. Srivastava A, Thomson S. Framework analysis: a qualitative methodology for applied policy research. Journal of Administration and Governance. 2009;4(2).

23. Mweli H. National framework for the teaching of reading in African languages in the foundation phase. Pretoria, Republic of South Africa: Department of Basic Education; 2020.

24. Brislin RW. Back-translation for cross-cultural research. Journal of Cross-Cultural Psychology. 1970;1(3):185-216.

25. Flaherty JA, Gaviria FM, Pathak D, Mitchell T, Wintrob R, Richman JA, et al. Developing instruments for cross-cultural psychiatric research. J Nerv Ment Dis. 1988;176(5):257-63.

26. Carter N, Bryant-Lukosius D, DiCenso A, Blythe J, Neville AJ. The use of triangulation in qualitative research. Oncol Nurs Forum. 2014;41(5):545-7.

27. Molyneux CS, Peshu N, Marsh K. Understanding of informed consent in a low-income setting: three case studies from the Kenyan Coast. Soc Sci Med. 2004;59(12):2547-59.

28. Molyneux S, Bull S. Consent and Community Engagement in Diverse Research Contexts: Reviewing and Developing Research and Practice:Participants in the Community Engagement and Consent Workshop, Kilifi, Kenya, March 2011. Journal of Empirical Research on Human Research Ethics. 2013;8(4):1-18.

29. Buyum AM, Kenney C, Koris A, Mkumba L, Raveendran Y. Decolonising global health: if not now, when? BMJ Glob Health. 2020;5(8).

30. Khan M, Abimbola S, Aloudat T, Capobianco E, Hawkes S, Rahman-Shepherd A. Decolonising global health in 2021: a roadmap to move from rhetoric to reform. BMJ Glob Health. 2021;6(3).

31. Chaudhry I, Thurtle V, Foday E, Leather AJM, Samai M, Wurie H, et al. Strengthening ethics committees for health-related research in sub-Saharan Africa: a scoping review. BMJ Open. 2022;12(11):e062847.

32. Boga M, Davies A, Kamuya D, Kinyanjui SM, Kivaya E, Kombe F, et al. Strengthening the Informed Consent Process in International Health Research through Community Engagement: The KEMRI-Wellcome Trust Research Programme Experience. PLOS Medicine. 2011;8(9):e1001089.

33. Beardmore-Gray A, Vousden N, Silverio SA, Charantimath U, Katageri G, Bellad M, et al. Planned early delivery for late preterm pre-eclampsia in a low- and middle-income setting: a feasibility study. Reprod Health. 2021;18(1):110.

For peer review only

Figure Legends

Figure 1: Thematic framework

Figure 2: Summary of recommended actions

For peer review only

For peer review only - http://bmjopen.bmj.com/site/about/guidelines.xhtml

Figure 1: Thematic framework

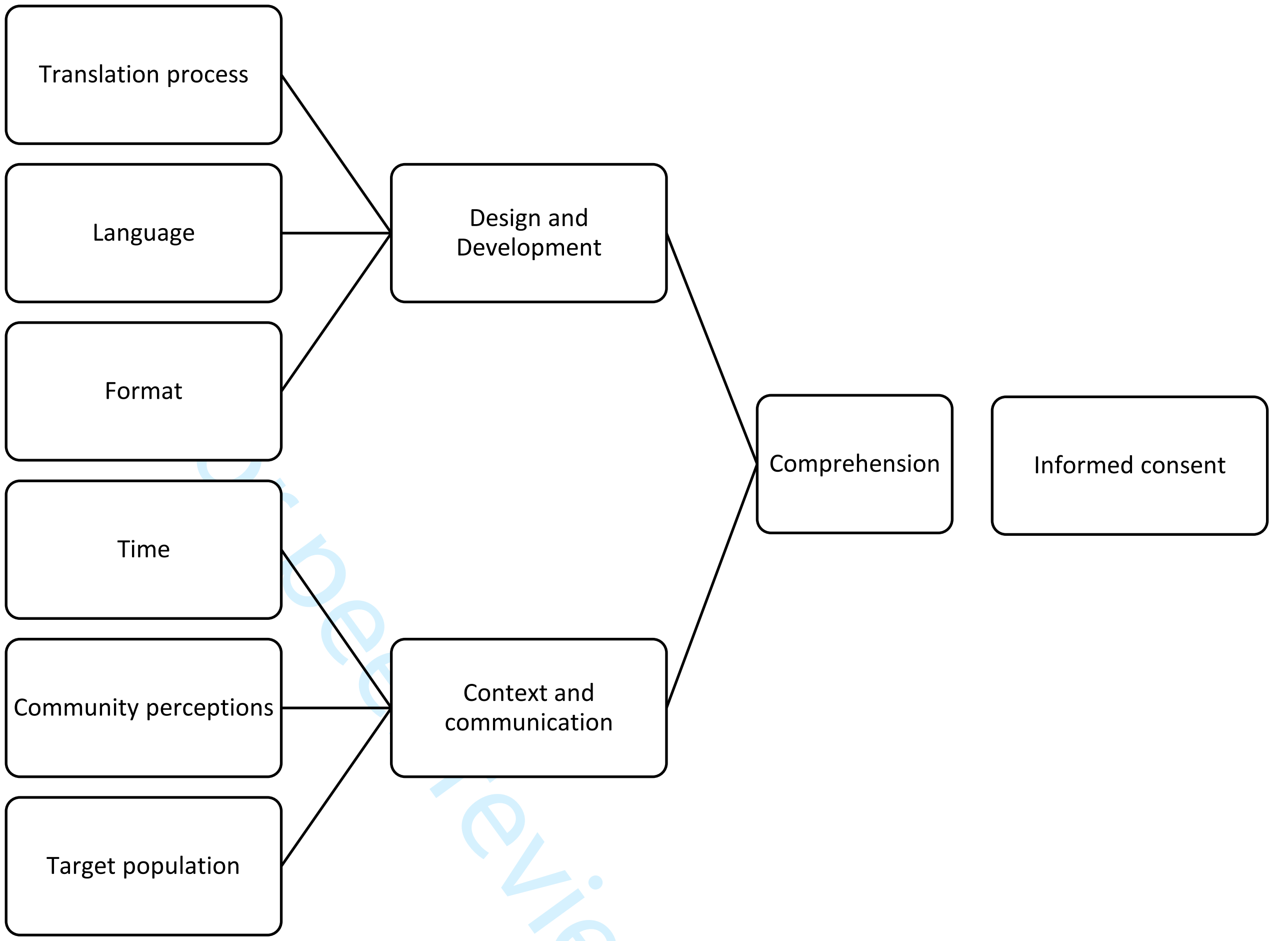

For peer review only

Figure 2: Summary of recommended actions

Research institutions and ethics committees

- Adopt a more flexible and adaptive approach to templates
- Suport research teams to develop recruitment materials that are context-specific
- Ensure study protocols allow sufficient time and funding to support a robust translation process and consent process including community engagement activities
- Ensure strong oversight mechanisms to verify the quality and appropriateness of translated materials
- Support further research into alternative methods of providing participant information, such as pictures and videos

Researchers

- Set aside sufficient time and funding to develop recruitment materials
- Meet face to face with translators and local language experts, ensuring the true meaning of recruitment materials can be understood
- Involve community representatives and local researchers from the outset, piloting early versions of translated materials and responding dynamically to feedback
- Move away from lengthy word documents with information that may be considered irrelevant by potential participants
- Consider a glossary of key terms at the start of any document, using simple and informal terms to explain important concepts or medical terms
- Consider the most appropriate format for the intended recipients, including flip charts and videos if appropriate

Translators

- Move away from literal, word for word translations
- Explore, and be guided by, local dialects and preferences for more informal language

Supplementary Figure 1: Theoretical framework

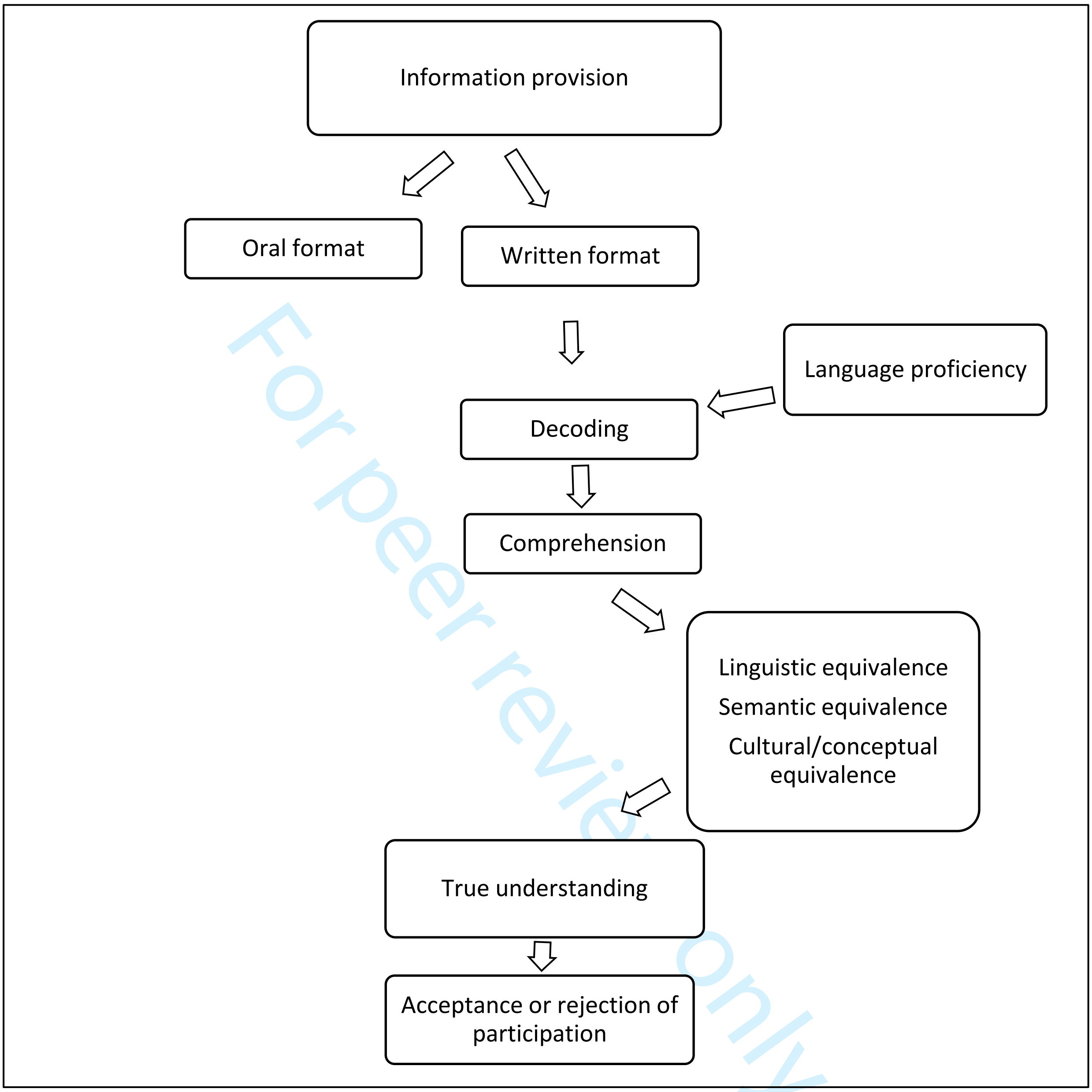

For peer review only

Lost in translation: supplementary material

Contents

Supplementary Table 1: Content analysis of recruitment documents 2
Supplementary Table 2: Interview topic guide 5
Supplementary Table 3: Suggested terms from focus group discussions 7

For peer review only

For peer review only - http://bmjopen.bmj.com/site/about/guidelines.xhtml

Supplementary Table 1: Content analysis of recruitment documents

Theme	Frequency (documents/13)
**Pregnancy specific**	
Pregnancy	6
Pre-eclampsia	5
Delivery	3
Childbirth	3
Placenta	2
Complication	4
Ultrasound	3
Urine sample	3
Umbilical cord	2
Adverse birth outcomes	1
Blood pressure	2
High blood pressure	3
Pregnant women	3
High blood pressure in pregnancy	1
Contractions	1
Planned early delivery	1
Pre-term birth	1
Protein level in the urine	1
**Participation**	
Take part	7
You are being invited to take part in a research project	4
Participation/Participating	10
Withdraw	6
You are free to decline to participate	1
You are free to withdraw from the study at any time	3
You are not forced to participate in this study	2
You do not have to take part in this research if you do not wish to do so	1
Your decision will not affect the affect the care you receive in any way	4
We would like to invite you to take part	1
There is no penalty if you choose not to take part	1
You can receive care whether you take part in our study or not	1
You do not have to be in the research study to receive health care	1

For peer review only - http://bmjopen.bmj.com/site/about/guidelines.xhtml

We request your co-operation	1
We would like to invite you to participate in this study	1
**Voluntary Consent**	
Your participation is entirely voluntary	9
Consent	6
Consent form	7
Permission	3
Informed choice	2
I consent voluntarily as a participant in this research	1
If you agree to take part	1
**Risk**	
Concerns	5
Dangerous	2
Risk	8
Serious complications/consequences	5
Side effects	4
Discomfort	5
No risk	4
Suffer	3
Problems	6
Risk factors	1
**Benefit**	
Benefit	6
Benefit others/Benefit to society	2
Benefits to you	1
Direct benefit/may not help you directly/no personal benefit	4
Improve/Improve care/Improve health/outcomes	4
Possible/potential benefits	6
Important benefits	1
**Healthcare care related**	
Your health/Your baby's health	5
Care	4
Symptoms and signs	2
Treatments/Treating	5
Severe problems	2
Samples	3
Results	4
Medicine	3
Injection	4

For peer review only - http://bmjopen.bmj.com/site/about/guidelines.xhtml

Infection	3
Condition	3
Drug/study drug	2
Health	1
Healthcare providers	2
Improve care	2
Swelling	2
Test	3
Swab	2
Health facilities	1
Health information	1
Healthcare	1
**Research concepts**	
What is the purpose of the study	9
Study	11
50/50 Chance	1
Academic collaborators	1
Analysis/Analysed	1
Chance	3
Collect	7
Research	10
Published/publications	3
Knowledge	3
Measure/measurements	3
Presentation at international meetings/conference	2
Sponsor	3
Title of research	1
**Confidentiality/data management**	
Anonymous/Anonymised	4
Confidential	11
Data	5
Kept under lock and key	1
Protected	5
Information	10
Information about you	5
Results	4
Personal information/details	4
Information that is collected about you and your baby	2
Identity	4
Contact information/details	3
Identify	2
Data collected about you	1
Data collection forms	1
Data manager	1

For peer review only - http://bmjopen.bmj.com/site/about/guidelines.xhtml

For peer review only

Supplementary Table 2: Interview topic guide

Details:

**Date**	
**Start time**	
**Finish time**	
**Location**	
**Initial(s) of participant(s)**	
**Age(s)**	
**Level of education**	
**Profession**	

Notes

**Example prompts**<br><br>*What does this term/phrase mean to you?*<br><br>*What do you understand by this term/phrase?*<br><br>*How might this term be translated into Nyanja/Bemba?*<br><br>*Is there an equivalent word?*<br><br>*Is there an equivalent concept?*<br><br>**If participant is a translator:**<br><br>*Can you tell me about your experience of translating research*	

For peer review only

materials from English to Nyanja/Bemba?<br><br>*Have you come across any challenges?*<br><br>*Is there anything you feel that may improve this process?*<br><br>**If participant is a researcher**:<br><br>*Can you tell me about your experience of using research materials when gaining consent?*<br><br>*Have you come across any challenges?*<br><br>*Is there anything you feel that may improve this process?*	

For peer review only

For peer review only - http://bmjopen.bmj.com/site/about/guidelines.xhtml

Supplementary Table 3: Suggested terms from focus group discussions

English	Nyanja
Pre-eclampsia	Bvuto la kutamanga kwamagazi ndikupezeka kwa dso kudya za mthupi mumitundo pa mene muzimai ali ndi pakati
Stroke	Sitoloko
Low birthweight	Mwana opepoka
Stillborn baby	Nthayo/makanda/ana
Premature baby	Mwana osakosa/osafikapo
Swelling of the legs	Kubvimba kwa mendo
Fitting	Kukunyuka
Urine	Mkhozo/mitundo
Protein in the urine	Kupezeka kwa zakudya zamthupi mu mitundo
High blood pressure	Kutamanga Kwamagazi (Bipi)
This research is brought to you by	Atibweretsera phunziro ndi…/ Akubweretserani…
What are the risks of taking part	Ciyopyezo kuipa/ Kodi kuipa kotengaku mbali ndi kwabwanji?
Research study	Maphunzilo/ Kufunafuna/ Kufufuza
Benefit	Ubwino/Phindu
What are the benefits of taking part?	Ubwino otengako mbali ndiwabwanji?
What is the purpose of the study	Colinga caphunziro ndi ciani?
Why have I been called/invited to take part?	Ndilifukwa ciani/ndaitanidwa kuti ndi tengeko mbali?
What will happen if I take part?	Kodi ndi ciani cizacitika ndi katengaka mbali
Consent form	Cipepala cobvomekeza
Randomisation	Magulu awiri/ Komputa iza zisankila/ Magulu losadzisankhira
Healthy	Umoyo wabwino
Address	Adelesi
Analyse results	Kusanda sanda
Problem/suffering	Mabvuto
The doctor will have to induce labour	Cilikidwa kuyambisidwa

For peer review only - http://bmjopen.bmj.com/site/about/guidelines.xhtml

Standards for Reporting Qualitative Research (SRQR)*

http://www.equator-network.org/reporting-guidelines/srqr/

Page/line no(s).

Title and abstract

**Title** - Concise description of the nature and topic of the study Identifying the study as qualitative or indicating the approach (e.g., ethnography, grounded theory) or data collection methods (e.g., interview, focus group) is recommended	1
**Abstract** - Summary of key elements of the study using the abstract format of the intended publication; typically includes background, purpose, methods, results, and conclusions	2-3

Introduction

**Problem formulation** - Description and significance of the problem/phenomenon studied; review of relevant theory and empirical work; problem statement	5
**Purpose or research question** - Purpose of the study and specific objectives or questions	6

Methods

**Qualitative approach and research paradigm** - Qualitative approach (e.g., ethnography, grounded theory, case study, phenomenology, narrative research) and guiding theory if appropriate; identifying the research paradigm (e.g., postpositivist, constructivist/ interpretivist) is also recommended; rationale**	9, Figures 1 & 2
**Researcher characteristics and reflexivity** - Researchers' characteristics that may influence the research, including personal attributes, qualifications/experience, relationship with participants, assumptions, and/or presuppositions; potential or actual interaction between researchers' characteristics and the research questions, approach, methods, results, and/or transferability	6-7, 18
**Context** - Setting/site and salient contextual factors; rationale**	6-8
**Sampling strategy** - How and why research participants, documents, or events were selected; criteria for deciding when no further sampling was necessary (e.g., sampling saturation); rationale**	7-8
**Ethical issues pertaining to human subjects** - Documentation of approval by an appropriate ethics review board and participant consent, or explanation for lack thereof; other confidentiality and data security issues	9
**Data collection methods** - Types of data collected; details of data collection procedures including (as appropriate) start and stop dates of data collection and analysis, iterative process, triangulation of sources/methods, and modification of procedures in response to evolving study findings; rationale**	6-8

For peer review only
For peer review only - http://bmjopen.bmj.com/site/about/guidelines.xhtml

**Data collection instruments and technologies** - Description of instruments (e.g., interview guides, questionnaires) and devices (e.g., audio recorders) used for data collection; if/how the instrument(s) changed over the course of the study	7-8, Supplementary Table 2
**Units of study** - Number and relevant characteristics of participants, documents, or events included in the study; level of participation (could be reported in results)	Table 1
**Data processing** - Methods for processing data prior to and during analysis, including transcription, data entry, data management and security, verification of data integrity, data coding, and anonymization/de-identification of excerpts	9-10
**Data analysis** - Process by which inferences, themes, etc., were identified and developed, including the researchers involved in data analysis; usually references a specific paradigm or approach; rationale**	9-10
**Techniques to enhance trustworthiness** - Techniques to enhance trustworthiness and credibility of data analysis (e.g., member checking, audit trail, triangulation); rationale**	10

Results/findings

**Synthesis and interpretation** - Main findings (e.g., interpretations, inferences, and themes); might include development of a theory or model, or integration with prior research or theory	10 - 16
**Links to empirical data** - Evidence (e.g., quotes, field notes, text excerpts, photographs) to substantiate analytic findings	Table 3

Discussion

**Integration with prior work, implications, transferability, and contribution(s) to the field** - Short summary of main findings; explanation of how findings and conclusions connect to, support, elaborate on, or challenge conclusions of earlier scholarship; discussion of scope of application/generalizability; identification of unique contribution(s) to scholarship in a discipline or field	16-18
**Limitations** - Trustworthiness and limitations of findings	18-19

Other

**Conflicts of interest** - Potential sources of influence or perceived influence on study conduct and conclusions; how these were managed	20
**Funding** - Sources of funding and other support; role of funders in data collection, interpretation, and reporting	10, 20

*The authors created the SRQR by searching the literature to identify guidelines, reporting standards, and critical appraisal criteria for qualitative research; reviewing the reference lists of retrieved sources; and contacting experts to gain feedback. The SRQR aims to improve the transparency of all aspects of qualitative research by providing clear standards for reporting qualitative research.

For peer review only

**The rationale should briefly discuss the justification for choosing that theory, approach, method, or technique rather than other options available, the assumptions and limitations implicit in those choices, and how those choices influence study conclusions and transferability. As appropriate, the rationale for several items might be discussed together.

Reference:

O'Brien BC, Harris IB, Beckman TJ, Reed DA, Cook DA. **Standards for reporting qualitative research: a synthesis of recommendations.** *Academic Medicine*, Vol. 89, No. 9 / Sept 2014 DOI: 10.1097/ACM.0000000000000388

For peer review only

For peer review only - http://bmjopen.bmj.com/site/about/guidelines.xhtml
